# Group sequential methods for interim monitoring of randomized clinical trials with time‐lagged outcome

**DOI:** 10.1002/sim.9580

**Published:** 2022-09-18

**Authors:** Anastasios A. Tsiatis, Marie Davidian

**Affiliations:** ^1^ Department of Statistics North Carolina State University Raleigh North Carolina

**Keywords:** augmented inverse probability weighting, early stopping, influence function, proportion of information

## Abstract

The primary analysis in two‐arm clinical trials usually involves inference on a scalar treatment effect parameter; for example, depending on the outcome, the difference of treatment‐specific means, risk difference, risk ratio, or odds ratio. Most clinical trials are monitored for the possibility of early stopping. Because ordinarily the outcome on any given subject can be ascertained only after some time lag, at the time of an interim analysis, among the subjects already enrolled, the outcome is known for only a subset and is effectively censored for those who have not been enrolled sufficiently long for it to be observed. Typically, the interim analysis is based only on the data from subjects for whom the outcome has been ascertained. A goal of an interim analysis is to stop the trial as soon as the evidence is strong enough to do so, suggesting that the analysis ideally should make the most efficient use of all available data, thus including information on censoring as well as other baseline and time‐dependent covariates in a principled way. A general group sequential framework is proposed for clinical trials with a time‐lagged outcome. Treatment effect estimators that take account of censoring and incorporate covariate information at an interim analysis are derived using semiparametric theory and are demonstrated to lead to stronger evidence for early stopping than standard approaches. The associated test statistics are shown to have the independent increments structure, so that standard software can be used to obtain stopping boundaries.

## 1 INTRODUCTION

In many randomized clinical trials, the primary analysis involves a comparison of two treatments, typically an active or experimental agent vs a control, which is formalized as inference on a scalar treatment effect parameter. When the primary outcome is a continuous measure, this parameter is usually the difference of treatment‐specific means. For a binary outcome, the treatment effect parameter may be the risk difference, risk ratio, or odds ratio; and the odds ratio under the assumption of a proportional odds model is often the treatment effect parameter of interest in trials involving an ordinal categorical outcome. The primary analysis is ordinarily based on a test statistic constructed using an estimator for the parameter of interest, for example, the difference of sample means or maximum likelihood (ML) estimator for the odds ratio in a proportional odds model. The overall sample size is established so that the power to detect a clinically meaningful departure from the hypothesis of no treatment effect at a given level of significance using the test statistic at the final analysis achieves some desired value, for example, 90%.

Most later‐stage clinical trials are monitored for the possibility of early stopping for efficacy or futility by a data and safety monitoring board (DSMB), with interim analyses planned at either fixed, predetermined analysis times or when specified proportions of the total “statistical information” to be gained from the completed trial have accrued.^1^ Ordinarily, at the time of an interim analysis, the test statistic to be used for the final analysis is computed based on the available data and compared to a suitable stopping boundary constructed to preserve the overall operating characteristics of the trial.^2^, ^3^

Because of staggered entry into the trial, the data available at the time of an interim analysis are from subjects who have already enrolled. Moreover, the primary outcome Y is ordinarily not known immediately but is ascertained after some lag time T, say. In some trials, the lag time T is the same for all participants, as in the case where Y is a continuous outcome that will be measured at a prespecified follow‐up time ${𝒯}_{F}$, for example, ${𝒯}_{F}=$ 1 year, so that $T={𝒯}_{F}$ for all subjects, and the treatment parameter is the difference in treatment means of Y at 1 year. Here, at the time of an interim analysis, Y will be available only for subjects enrolled for at least 1 year, so that the analysis can be based only on the data for these subjects.

In other settings, the time lag T may be different for different participants and, moreover, T may be correlated with the outcome Y. This issue arises in many clinical trials of COVID‐19 therapeutics conducted by the Accelerating COVID‐19 Therapeutic Interventions and Vaccines (ACTIV) public‐private partnership. In an ongoing clinical trial coordinated through the ACTIV‐3b: Therapeutics for Severely Ill Inpatients with COVID‐19 (TESICO) master protocol,^4^ patients hospitalized with acute respiratory distress syndrome are randomized to receive an active agent or placebo and followed for up to ${𝒯}_{F}=90$ days. The primary outcome Y is an ordinal categorical variable with six levels. The first five categories reflect a subject's status at 90 days following enrollment: (1) at home and off oxygen for at least 77 days (the most favorable category); (2) at home and off oxygen for at least 49 but no more than 76 days; (3) at home and off oxygen for at least 1 but no more than 48 days; (4) not hospitalized and either at home on oxygen or receiving care elsewhere; and (5) still hospitalized or in hospice care. Category 6 (the worst) corresponds to death within the 90 day follow‐up period. While Categories 1‐5 cannot be ascertained until a subject has been followed for the full 90 days, that a subject's outcome is Category 6 is known at the time of death. Thus, the time lag before ascertainment is $T={𝒯}_{F}=90$ days for subjects with Y=1,…,5 and is equal to the (random) time of death $T\leq {𝒯}_{F}=90$ for those with Y=6. In TESICO, the treatment effect parameter is the odds ratio for active agent relative to placebo under an assumed proportional odds model. Similarly, in a clinical trial coordinated through the ACTIV‐2: a study for outpatients with COVID‐19 master protocol (Study A5401),^5^ subjects within seven days of self‐reported COVID‐19 onset are randomized to receive an active agent or placebo and followed for up to ${𝒯}_{F}=28$ days for the binary outcome Y, where Y=1 if the subject dies or is or hospitalized within 28 days and Y=0 otherwise. For subjects who die or are hospitalized at time T prior to 28 days, Y=1 is ascertained after a time lag $T\leq {𝒯}_{F}=28$, whereas that Y=0 can be ascertained only after the full 28 days, and $T={𝒯}_{F}=28$. Here, the treatment parameter is the relative risk (risk ratio) of hospitalization/death for active agent vs placebo.

At the time of an interim analysis in TESICO and A5401, the available data include the outcomes for all enrolled subjects who have been followed for at least 90 and 28 days, so for whom $T={𝒯}_{F}=90$ or 28, respectively, along with the outcomes for enrolled subjects who do not have 90 or 28 days of follow up but have already been observed to die (Y=6) in TESICO or to be hospitalized or die (Y=1) in A5401 ($T\leq {𝒯}_{F}=90$ or 28). Thus, information on Category 6 in TESICO will accumulate more rapidly than that on the other categories; similarly, information on hospitalization/death in A5401 will accrue more quickly than information on subjects who remain alive and unhospitalized at day 28. Intuitively, basing an interim analysis on all observed outcomes will naively overrepresent Y=6 and Y=1 and lead to potentially biased inference on the treatment effect parameters.

To characterize this issue more precisely, if C is the time from a subject's entry into the study to the time of an interim analysis, then Y is known at the time of an interim analysis if C>T. Otherwise, the time lag T for ascertainment of Y is censored at C, and Y is not observed. Basing the analysis on all subjects with C>T, the “complete cases” for whom Y is observed, without taking appropriate account of the fact that Y is not available for those with C≤T leads to the bias noted above. This bias arises because subjects with shorter lag times T are more likely to be represented among the complete cases than those subjects with longer lag times, and because T and Y may be correlated, as in TESICO and A5401, the distribution of Y among the complete cases thus will not reflect the true distribution of Y. These considerations suggest that a valid interim analysis can be obtained by using only the data from enrolled subjects followed for the full, maximum follow‐up period ${𝒯}_{F}$, that is, for whom $C\geq {𝒯}_{F}$. In studies like those above involving a continuous outcome or ordinal categorical outcome, as in TESICO, the standard interim analysis is based on the estimator to be used at the final analysis using only the data on subjects with $C\geq {𝒯}_{F}$, as there is no apparent general approach to “adjusting” for the censoring. In the case of a binary outcome as in A5401, the standard interim analysis does use the information on censoring; for example, if the treatment effect is the risk ratio, the estimator is the ratio of the treatment‐specific Kaplan‐Meier estimators for the probability of death or hospitalization at ${𝒯}_{F}=28$ days.

A goal of an interim analysis is to stop the trial as early as possible if there is sufficiently strong evidence to do so. It is thus natural to consider whether or not it is possible to make more efficient use of the available data at the time of an interim analysis to enhance precision and thus the strength of the evidence for stopping. One step toward increasing efficiency of interim analyses would be a general approach to accounting for censoring for any outcome and treatment effect parameter that incorporates data from all of the complete cases, not just those with $C\geq {𝒯}_{F}$. In addition, it may be possible to incorporate partial information; for example, in TESICO, a subject who is at day 45 since study entry and still in the hospital at the time of an analysis, so for whom $C=45<{𝒯}_{F}=90$, can end up only in Categories 3‐6, so have Y=3,4,5, or 6, at 90 days. There may be baseline covariates as well as intermediate measures of the outcome or other post‐treatment variables that also could be exploited to increase precision at an interim analysis.

In this article, we propose a general group sequential framework for clinical trials with a possibly censored, time‐lagged outcome, which leads to practical strategies for interim monitoring. Treatment effect estimators are proposed via application of semiparametric theory,^6^, ^7^ which dictates how censoring can be taken into account and baseline and time‐dependent covariate information can be exploited in a principled way to increase precision and thus yield stronger evidence for early stopping. Although ordinarily incorporation of post‐randomization covariates in clinical trial analyses, for example, in models for covariate adjustment, raises concerns over causal interpretations, the use of such information in the proposed approach follows from semiparametric theory and serves only to increase efficiency. Estimation of the risk ratio via treatment‐specific Kaplan‐Meier estimators as described above emerges as a simple special case, which can be improved upon through incorporation of covariates. We show that the test statistics based on these estimators have an independent increments structure,^8^ which allows standard software for constructing stopping boundaries^2^, ^3^, ^9^ to be used. Two interim monitoring strategies are discussed: an information‐based monitoring approach under which the trial will continue, with possibly a larger sample size than originally planned, until the full, target statistical information accrues; and a fixed‐sample size approach appropriate in settings where the planned sample size cannot be increased due to resource and other constraints. We focus on the common case of two treatments; extension to more than two treatments is possible^10^ and could be adapted to group sequential methods for multi‐arm trials.^11^, ^12^

In Section 2, we introduce the basic statistical framework and assumptions, and we sketch the estimation approach and state the independent increments property in Section 3. In Section 4, we describe practical implementation of the resulting approach to interim monitoring. We demonstrate the performance of the methods in a series of simulation studies in Section 5, and we present a case study exemplifying the use of the methods for a simulated trial based on TESICO. Technical details and sketches of proofs of results are given in the Supplemental Material.

## 2 STATISTICAL FRAMEWORK

### 2.1 General model

As in Section 1, denote the outcome by Y. Let A denote the treatment indicator, where A=0 (1) corresponds to control (active/experimental treatment), and π=pr(A=1) is the probability of being assigned to active treatment; and let X be a vector of baseline covariates. Treatment effects are often characterized in terms of a model for (features of) the distribution of (Y,A) or (Y,A,X), which involves parameters $\left(\alpha^{T},\beta \right)$, where β is the scalar treatment effect parameter of interest and α is a vector of nuisance parameters, and the model is parameterized such that β=0 corresponds to the null hypothesis of no treatment effect.

In the case of the first example in Section 1 of continuous Y, β=E(Y|A=1)−E(Y|A=0); equivalently,

(1) E(Y|A=a)=α+βa,a=0,1.

For an ordinal categorical outcome with c categories, as in TESICO with c=6, the outcome can be represented as either a scalar random variable Y taking values 1,…,c or a random vector Y={I(Cat=1),…,I(Cat=c−1)}, where Cat takes values 1,…,c. Using the first definition, the treatment effect can be defined through an assumed proportional odds model

(2) $$\text{logit}\{\mathrm{pr}(Y\leq j|A=a)\}=\alpha_{j}+\beta a,\;j=1,\ldots ,c-1,\;\alpha ={\left(\alpha_{1},\ldots ,\alpha_{c-1}\right)}^{T},\;a=0,1,$$

where logit(p)=log{p/(1−p)}, so that β is the log odds ratio of interest. If the conditional (on X) treatment effect is of interest, replace (2) by $\text{logit}\{\mathrm{pr}(Y\leq j|X=x,A=a)\}=\alpha_{j}+\beta a+\xi^{T}x$, where now $\alpha ={\left(\alpha_{1},\ldots ,\alpha_{c-1},\xi^{T}\right)}^{T}$. If Y is binary as in A5401 and the relative risk (risk ratio) pr(Y=1|A=1)/pr(Y=1|A=0) is the focus, taking

(3) E(Y|A=a)=exp(α+βa),a=0,1,

corresponds to log relative risk β.

In general, estimators for the parameter of interest β in (1)‐(3) and other models based on the data available at the final analysis, at which time (Y,A,X) are known for all n participants, are obtained by solving, jointly in α and β, appropriate estimating equations. Namely, with independent and identically distributed (iid) data $\left(Y_{i},A_{i},X_{i}\right)$, i=1,…,n, available, and p equal to the dimension of ${\left(\alpha^{T},\beta \right)}^{T}$, $\hat{\alpha }$ and $\hat{\beta }$ solve in α and β equations of the form

(4) $$\overset{n}{\underset{i=1}{\sum }}ℳ(Y_{i},A_{i},X_{i};\alpha ,\beta )=0,$$

where ℳ(Y,A,X;α,β) is a p‐dimensional vector of functions such that $E\{ℳ(Y,A,X;\alpha_{0},\beta_{0})\}=0$, and $\alpha_{0}$ and $\beta_{0}$ are the true values of α and β under the assumption that the model is correctly specified. For example, under models (1) and (3)

(5) $$\text{(a)}\;ℳ(Y,A,X;\alpha ,\beta )=\left(\begin{array}{c} 1\\ A \end{array}\right)(Y-\alpha -\beta A)\;\text{and}\;\text{(b)}\;ℳ(Y,A,X;\alpha ,\beta )=\left(\begin{array}{c} 1\\ A \end{array}\right)\{Y-\exp(\alpha +\beta A)\},$$

respectively. Writing ${\bar{Y}}_{a}=\sum_{i=1}^{n}Y_{i}I(A_{i}=a)/\sum_{i=1}^{n}I(A_{i}=a)$, a=0,1, the treatment‐specific sample means, the estimator obtained from (5a) is $\hat{\beta }={\bar{Y}}_{1}-{\bar{Y}}_{0}$ and that from (5b) is $\hat{\beta }=\log({\bar{Y}}_{1}/{\bar{Y}}_{0})$, which is the estimator for the relative risk used in A5401. Under model (2), with $\text{expit}(u)=e^{u}/(1+e^{u})$,

(6) $$ℳ(Y,A,X;\alpha ,\beta )=𝒟(A)\left(\begin{array}{c} I(Y\leq 1)-\text{expit}(\alpha_{1}+\beta A)\\ \vdots \\ I(Y\leq c-1)-\text{expit}(\alpha_{c-1}+\beta A) \end{array}\right),$$

where 𝒟(A) is a (c × c−1) matrix of functions of A; the ML estimator^13^ takes $𝒟(A)=D^{T}(A;\alpha ,\beta )V^{-1}(A;\alpha ,\beta )$, where D(A;α,β) is the (c−1×c) gradient matrix of the vector ${\left\{I(Y\leq 1)-\text{expit}(\alpha_{1}+\beta A),\ldots ,I(Y\leq c-1)-\text{expit}(\alpha_{c-1}+\beta A)\right\}}^{T}$ in (6) with respect to $\alpha_{1},\ldots ,\alpha_{c-1},\beta$, and V(A;α,β) is its (c−1×c−1) conditional covariance matrix given A.

In general, given a particular model and estimating equations defined by the corresponding function ℳ(Y,A,X;α,β), let 𝒢(α,β) be the last row of the (p×p) matrix

(7) $$-{\left[E\left\{\frac{\partial ℳ(Y,A,X;\alpha ,\beta )}{\partial \alpha^{T}\partial \beta }\right\}\right]}^{-1},$$

where the matrix inside the expectation is the (p×p) matrix of partial derivatives of the p components of ℳ(Y,A,X;α,β) with respect to ${\left(\alpha^{T},\beta \right)}^{T}$. Then, with $𝒢(\alpha_{0},\beta_{0})$ denoting this expression evaluated at $\alpha_{0},\beta_{0}$,

(8) $$m(Y,A,X;\alpha_{0},\beta_{0})=𝒢(\alpha_{0},\beta_{0})ℳ(Y,A,X;\alpha_{0},\beta_{0}),$$

is referred to as the influence function of the corresponding estimator for β and has mean zero. From the theory of M‐estimation^14^ and semiparametric theory,^7^ it can be shown the estimator $\hat{\beta }$ obtained by solving in β the estimating equation

(9) $$\overset{n}{\underset{i=1}{\sum }}m(Y,A,X;\hat{\alpha },\beta )=0,$$

where $\hat{\alpha }$ is any root‐n consistent estimator for α, has influence function (8). Tsiatis et al^10^ show this explicitly in the case of (6). Such estimators are consistent for the true value $\beta_{0}$ and asymptotically normal, where the variance of the limiting normal distribution of $n^{1/2}(\hat{\beta }-\beta_{0})$ is equal to $\mathrm{var}\{m(Y,A,X;\alpha_{0},\beta_{0})\}=E\{m{\left(Y,A,X;\alpha_{0},\beta_{0}\right)}^{2}\}$, so that approximate (large sample) SEs and test statistics are readily derived.

From semiparametric theory,^7^ there is a one‐to‐one correspondence between influence functions and estimators. Thus, if the form of influence functions in a specific model involving a parameter β can be derived, estimating equations leading to estimators for β can be developed. As we demonstrate in Section 3, influence functions corresponding to estimators for β based on the data available at an interim analysis can be derived from the influence function (8), and the resulting estimators exploit baseline and time‐dependent covariate information to gain precision.

### 2.2 Data and assumptions

To characterize the data that would be available at an interim analysis, we first describe more fully the data that would be available at the final analysis if the trial were carried out to completion. Subjects enter the trial in a staggered fashion; thus, if the trial starts at calendar time 0, denote by E the calendar time at which a subject enters the trial. As in Section 1, let T denote the time lag in ascertaining the outcome Y; thus, T is the time since entry at which Y is determined, measured on the scale of subject time. We assume that Y can be determined with certainty by the maximum follow‐up period ${𝒯}_{F}$ for any subject, so that $\mathrm{pr}(T\leq {𝒯}_{F})=1$. In addition to baseline covariates X, time‐dependent covariate information may be collected on each participant up to the time Y is ascertained. Denote by L(u) the vector of such information at time u following entry into the study, and let $\bar{L}(u)=\{L(s):0\leq s\leq u\}$ be the history of the time‐dependent covariate information through time u. Thus, $\bar{L}(T)$ represents the covariate history for a subject for whom Y is ascertained after time lag T.

With these definitions, for a trial with planned total sample size n, the data available at the final analysis are iid

(10) $$\{E_{i},X_{i},A_{i},T_{i},Y_{i},{\bar{L}}_{i}(T_{i})\},\;i=1,\ldots ,n;$$

we refer to (10) as the full data. As in Section 2.1, estimation of β at the final analysis is based only on the data on Y, A, and possibly X (in the case of conditional inference), and E, T, and $\bar{L}(T)$ are not used, and we call (8) a full data influence function.

Now consider the data that would be available at an interim analysis at calendar time t following the start of the trial at calendar time 0. It proves convenient for the developments in Section 3 to represent these data in terms of the full data (10) that would be available at the final analysis were the trial to be carried out to completion. At t, data will be observed only for subjects for whom E≤t. For such subjects, define C(t)=t−E to be the censoring time, that is, the time from a participant's entry into the study to the time of the interim analysis. If the time lag T a subject would have in ascertaining the outcome is such that T≤C(t), then Y would be available at t; otherwise, Y would not yet be observed. Accordingly, define U(t)=min{T,C(t)} and Δ(t)=I{T≤C(t)}, so that Y is available at the time of the interim analysis only if Δ(t)=1. With these definitions, the data available at an interim analysis at calendar time t can be represented as iid

(11) $${𝒪}_{i}^{\left(t\right)}=I(E_{i}\leq t)\left[\;E_{i},X_{i},A_{i},U_{i}(t),\Delta_{i}(t),\Delta_{i}(t)Y_{i},{\bar{L}}_{i}\{U_{i}(t)\}\;\right],\;i=1,\ldots ,n,$$

where then $n(t)=\sum_{i=1}^{n}I(E_{i}\leq t)$ is the number of subjects of the n planned enrolled in the trial by calendar time t.

As noted in Section 1, an interim analysis that uses all of the available data, including those from subjects for whom $T<{𝒯}_{F}$, can naively overrepresent some values of the outcome over others. In terms of (11), the data on which this naive analysis would be based involve only subjects i who are enrolled and whose outcome is available, that is, for whom $I\{E_{i}\leq t,\Delta_{i}(t)=1\}=1$. In contrast, a valid analysis that uses only the data from subjects enrolled for at least the full, maximum follow‐up period ${𝒯}_{F}$ involves subjects i for whom $I\{E_{i}\leq t,C_{i}(t)\geq {𝒯}_{F}\}=1$. In the next section, we appeal to semiparametric theory as noted at the end of Section 2.1 to deduce methods yielding valid inference on β based on the available data (11) that can improve substantially on this analysis and thus lead to more efficient interim analyses.

## 3 INFERENCE BASED ON INTERIM DATA

### 3.1 Treatment effect estimation

We first present general estimating equations using the available data (11) at an interim analysis at time t that yield treatment effect estimators offering gains in precision relative to the estimator based only on subjects for whom $I\{E_{i}\leq t,C_{i}(t)\geq {𝒯}_{F}\}=1$. Letting “╨” denote statistical independence, assume that X╨A, which is guaranteed by randomization. Also assume that

(12) $$E╨\{X,A,T,Y,\bar{L}(T)\};$$

(12) implies that subjects enter according to a completely random process, which is reasonable in many trials. Because C(t)=t−E, (12) also implies that $C(t)╨\{X,A,T,Y,\bar{L}(T)\}$. We discuss weakening these assumptions in Section 7. We also require that $\mathrm{pr}\{C(t)>{𝒯}_{F}\}>0$, so that there is positive probability of seeing subjects for whom the final outcome has been ascertained at an interim analysis at t and so that the first interim analysis must occur at least ${𝒯}_{F}$ time units after the start of the trial.

We first summarize the theoretical underpinnings of the practical, more efficient interim monitoring approach we propose in Section 4. Under the above assumptions, if $m(Y,A,X;\alpha_{0},\beta_{0})$ is the influence function of a given estimator for a treatment effect parameter β in a model for (Y,A,X) as in Section 2.1, so based on the full data (10), then semiparametric theory yields that influence functions for estimators for β based on the available data ${𝒪}^{\left(t\right)}$ in (11) at an interim analysis at time t are of the form

(13) $$\begin{array}{ll} & \frac{I(E\leq t)}{\mathrm{pr}(E\leq t)}\left(\frac{\Delta (t)m(Y,A,X;\alpha_{0},\beta_{0})}{{𝒦}_{t}\{U(t)\}}+\int_{0}^{t}\frac{dM_{c}^{\left(t\right)}(u)\mu (m,u;\alpha_{0},\beta_{0})}{{𝒦}_{t}(u)}\right.\\ & \;-(A-\pi )f(X)+\left.\int_{0}^{t}dM_{c}^{\left(t\right)}(u)\left[h\{u,X,A,\bar{L}(u)\}-\mu (h,u)\right]\right). \end{array}$$

In (13), f(X) is an arbitrary function of X; $h\{u,X,A,\bar{L}(u)\}$ is an arbitrary function of u, X, A, and $\bar{L}(u)$; and

$${𝒦}_{t}(u)=\mathrm{pr}\{C(t)\geq u|E\leq t\},\;\mu (m,u;\alpha_{0},\beta_{0})=E\{m(Y,A,X;\alpha_{0},\beta_{0})|T\geq u\},\;\mu (h,u)=E\left[h\{u,X,A,\bar{L}(u)\}|T\geq u\right],$$

$$dM_{c}^{\left(t\right)}(u)=dN_{c}^{\left(t\right)}(u)-d\Lambda_{c}^{\left(t\right)}(u){𝒴}^{\left(t\right)}(u),\;N_{c}^{\left(t\right)}(u)=I\{U(t)\leq u,\Delta (t)=0\},\;{𝒴}^{\left(t\right)}(u)=I\{U(t)\geq u\},\;\Lambda_{c}^{\left(t\right)}(u)=-\log\{{𝒦}_{t}(u)\}.$$

Here, ${𝒦}_{t}(u)$ is the survival distribution for the censoring variable C(t) at the time of the interim analysis, $N_{c}^{\left(t\right)}(u)$ and ${𝒴}^{\left(t\right)}(u)$ are the censoring counting process and at‐risk process, and $\Lambda_{c}^{\left(t\right)}(u)$ is the cumulative hazard function for censoring.

Let ${\hat{𝒦}}_{t}(u)$ be the Kaplan‐Meier estimator for ${𝒦}_{t}(u)$ using the data $\left\{U_{i}(t),1-\Delta_{i}(t)\right\}$ for i such that $E_{i}\leq t$, and define ${\hat{\Lambda }}_{c}^{\left(t\right)}(u)=-\log\{{\hat{𝒦}}_{t}(u)\}$, $d{\hat{M}}_{ci}^{\left(t\right)}(u)=dN_{ci}^{\left(t\right)}(u)-d{\hat{\Lambda }}_{c}^{\left(t\right)}(u){𝒴}_{i}^{\left(t\right)}(u)$, and ${\hat{\pi }}_{t}=\sum_{i=1}^{n}I(E_{i}\leq t)A_{i}/n(t)$, the proportion of enrolled subjects at t assigned to active treatment. Then it can be shown that estimating equations corresponding to the influence functions in (13) based on the available data (11) yielding estimators for β are of the form

(14) $$\overset{n}{\underset{i=1}{\sum }}I(E_{i}\leq t)\left[\frac{\Delta_{i}(t)m\{Y_{i},A_{i},X_{i};\hat{\alpha }(t),\beta \}}{{\hat{𝒦}}_{t}\{U_{i}(t)\}}-(A_{i}-{\hat{\pi }}_{t})f(X_{i})+\int_{0}^{t}d{\hat{M}}_{ci}^{\left(t\right)}(u)h\{u,X_{i},A_{i},{\bar{L}}_{i}(u)\}\right]=0,$$

where $\hat{\alpha }(t)$ is a consistent estimator for α based on the available data at t. For a specific model, corresponding full data influence function $m(Y,A,X;\alpha_{0},\beta_{0})$, and choice of the functions f(X) and $h\{u,X,A,\bar{L}(u)\}$, to be discussed momentarily, an estimator for β based on the data available at interim analysis time t is the solution to (14).

Taking f(X)≡0 and $h\{u,X,A,\bar{L}(u)\}\equiv 0$ in (14) yields the estimating equation

(15) $$\overset{n}{\underset{i=1}{\sum }}I(E_{i}\leq t)\left[\frac{\Delta_{i}(t)m\{Y_{i},A_{i},X_{i};\hat{\alpha }(t),\beta \}}{{\hat{𝒦}}_{t}\{U_{i}(t)\}}\right]=0,$$

whose solution is a so‐called inverse probability weighted complete case (IPWCC) estimator, which effectively bases estimation of β on only subjects for whom Y is available at t, that is, the complete cases at t, but with inverse weighting by the censoring distribution “adjusting” appropriately for the lag time in ascertaining the outcome. In particular, intuitively, the inverse weighting of the complete cases by the probability of being represented in the available data accounts for the fact that subjects with shorter lag times are more likely to be represented, so that the weighted sample of completers mimics the distribution of Y if there were no censoring. Judicious nonzero choices of f(X) and $h\{u,X,A,\bar{L}(u)\}$ facilitate exploiting baseline and time‐dependent covariate information to gain efficiency over the IPWCC estimator solving (15) through the two rightmost “augmentation” terms in the bracketed expression in (14), leading to what is referred to as an augmented inverse probability weighted complete case (AIPWCC) estimator for β; the optimal such choices are discussed below.

A counterintuitive result from semiparametric theory is that, for any arbitrary f(X) and $h\{u,X,A,\bar{L}(u)\}$, it is possible to improve the precision of the above estimators by replacing the Kaplan‐Meier estimator ${\hat{𝒦}}_{t}(u)$ by treatment‐specific Kaplan‐Meier estimators ${\hat{𝒦}}_{t}(u,a)$, say, obtained using the data $\left\{U_{i}(t),1-\Delta_{i}(t)\right\}$ for i such that $E_{i}\leq t$ and $A_{i}=a$, a=0,1, even though because of (12) the distribution of C(t) is not treatment dependent. This substitution leads to influence functions for estimators for β based on the available data of the form

(16) $$\begin{array}{ll} & \frac{I(E\leq t)}{\mathrm{pr}(E\leq t)}\left(\frac{\Delta (t)m(Y,A,X;\alpha_{0},\beta_{0})}{{𝒦}_{t}\{U(t),A\}}+\int_{0}^{t}\frac{dM_{c}^{\left(t\right)}(u,A)\mu (m,u,A;\alpha_{0},\beta_{0})}{{𝒦}_{t}(u,A)}\right.\\ & \;-(A-\pi )f(X)+\left.\int_{0}^{t}dM_{c}^{\left(t\right)}(u,A)\left[h\{u,X,A,\bar{L}(u)\}-\mu (h,u,A)\right]\right), \end{array}$$

where now

$$\begin{array}{cc} & {𝒦}_{t}(u,A)=\mathrm{pr}\{C(t)\geq u|E\leq t,A\},\mu (m,u,A;\alpha_{0},\beta_{0})=E\{m(Y,A,X\alpha_{0},\beta_{0})|T\geq u,A\},\\ & \mu (h,u,A)=E\left[h\{u,X,A,\bar{L}(u)\}|T\geq u,A\right], \end{array}$$

$$dM_{c}^{\left(t\right)}(u,A)=dN_{c}^{\left(t\right)}(u)-d\Lambda_{c}^{\left(t\right)}(u,A){𝒴}^{\left(t\right)}(u),\;\Lambda_{c}^{\left(t\right)}(u,A)=-\log\{{𝒦}_{t}(u,A)\}.$$

Estimating equations corresponding to (16) are then

(17) $$\overset{n}{\underset{i=1}{\sum }}I(E_{i}\leq t)\left[\frac{\Delta_{i}(t)m\{Y_{i},A_{i},X_{i};\hat{\alpha }(t),\beta \}}{{\hat{𝒦}}_{t}\{U_{i}(t),A_{i}\}}-(A_{i}-{\hat{\pi }}_{t})f(X_{i})+\int_{0}^{t}d{\hat{M}}_{ci}^{\left(t\right)}(u,A_{i})h\{u,X_{i},A_{i},{\bar{L}}_{i}(u)\}\right]=0,$$

where now ${\hat{\Lambda }}_{c}^{\left(t\right)}(u,a)=-\log\{{\hat{𝒦}}_{t}(u,a)\}$; and $d{\hat{M}}_{ci}^{\left(t\right)}(u,a)=dN_{ci}^{\left(t\right)}(u)-d{\hat{\Lambda }}_{c}^{\left(t\right)}(u,a){𝒴}_{i}^{\left(t\right)}(u)$. The estimating equations (17) with f(X)≡0 and $h\{u,X,A,\bar{L}(u)\}\equiv 0$,

(18) $$\overset{n}{\underset{i=1}{\sum }}I(E_{i}\leq t)\left[\frac{\Delta_{i}(t)m\{Y_{i},A_{i},X_{i};\hat{\alpha }(t),\beta \}}{{\hat{𝒦}}_{t}\{U_{i}(t),A_{i}\}}\right]=0,$$

yield an IPWCC estimator, and, again, nonzero choices of f(X) and $h\{u,X,A,\bar{L}(u)\}$ lead to an AIPWCC estimator.

When Y is a binary outcome, as in study A5401, it can be shown that the IPWCC estimator $\hat{\beta }(t)$ solving (18) is algebraically identical to the logarithm of the ratio of treatment‐specific Kaplan‐Meier estimators for the probability of death or hospitalization at ${𝒯}_{F}$ days. Thus, as noted in Section 1, the standard estimator for the risk ratio at an interim analysis is a special case of the general formulation here. Moreover, because this estimator is equivalent to an IPWCC estimator, it should be possible to obtain more efficient inference on the risk ratio at an interim analysis via an AIPWCC estimator.

Semiparametric theory provides the optimal choices of f(X) and $h\{u,X,A,\bar{L}(u)\}$ yielding the most precise AIPWCC estimator solving either of (14) or (17), given by

(19) $$\begin{array}{ll} f^{opt}(X) & =E\{m(Y,A,X;\alpha_{0},\beta_{0})|X,A=1)-E\{m(Y,A,X;\alpha_{0},\beta_{0})|X,A=0),\\ h^{opt}\{u,X,A,\bar{L}(u)\} & =\frac{E\{m(Y,A,X;\alpha_{0},\beta_{0})|T\geq u,X,A,\bar{L}(u)\}}{{𝒦}_{t}(u)}. \end{array}$$

The conditional expectations in (19) are not likely to be known in practice. We propose an approach to approximating $f^{opt}(X)$ and $h^{opt}\{u,X,A,\bar{L}(u)\}$ in Section 4. We recommend estimating β at an interim analysis at time t by $\hat{\beta }(t)$ solving an estimating equation of the form (17) with the approximations for $f^{opt}(X)$ and $h^{opt}\{u,X,A,\bar{L}(u)\}$ substituted.

From semiparametric theory, estimators solving estimating equations of the form (14) or (17) are consistent for $\beta_{0}$ (for n(t) and n large) and asymptotically normal, where, as at the end of Section 2.1, the variance of the large sample distribution of $\hat{\beta }(t)$ can be obtained from the variance of the corresponding influence function. Thus, the resulting approximate SEs $SE\{\hat{\beta }(t)\}$ can be used to form a Wald‐type test statistic, $T(t)=\hat{\beta }(t)/SE\{\hat{\beta }(t)\}$ appropriate for addressing the null hypothesis of no treatment effect, $H_{0}:\beta_{0}=0$.

We conclude this section by noting an important implication of these results. In the case where the full data (10) are available, as would be the case at the conclusion of the trial if not stopped early, the preceding developments lead to covariate‐adjusted estimators for β based on the full data that have the potential to yield increased efficiency over the usual full data analyses outlined in Section 2.1. In particular, considering (17), if $t_{end}$ is the calendar time at which the trial concludes with the full data accrued and outcomes for all subjects ascertained, $I(E_{i}\leq t_{end})=1$, $\Delta_{i}(t_{end})=1$, ${𝒦}_{t_{end}}\{U_{i}(t_{end}),A_{i}\}=1$, and $d{\hat{M}}_{ci}^{\left(t_{end}\right)}(u,A_{i})=0$, i=1,…,n, and (17) becomes

(20) $$\overset{n}{\underset{i=1}{\sum }}\left[m\{Y_{i},A_{i},X_{i};\hat{\alpha }(t_{end}),\beta \}-(A_{i}-\hat{\pi })f(X_{i})\right]=0,$$

where $\hat{\pi }=n^{-1}\sum_{i=1}^{n}A_{i}$, with corresponding influence function

(21) $$m(Y,A,X;\alpha_{0},\beta_{0})-(A-\pi )f(X).$$

As above, the optimal choice of f(X) leading to the most precise estimator solving (20) is that given in (19). The estimating Equation (20) is of the form of those in Zhang et al^15^ Thus, the proposed approach leads naturally to estimators for a final analysis that exploit baseline covariate information to improve efficiency through the “augmentation term” (A−π)f(X).

### 3.2 Interim analysis

In practice, interim analyses will be carried out at times $t_{1}<\cdots <t_{K}$, with the possibility of stopping the trial early, for example, for efficacy if evidence of a large treatment effect emerges at an interim analysis. That is, focusing on efficacy, the trial may be stopped at the first interim analysis time at which the relevant test statistic exceeds some appropriate stopping boundary; that is, if

$$|T(t_{j})|\geq b_{j},\;j=1,\ldots ,K,$$

for a two‐sided alternative or $T(t_{j})\geq$ or $\leq b_{j}$, j=1,…,K, for a one‐sided alternative, where $b_{j}$, j=1,…,K, are the stopping boundaries. As is well‐studied in the group sequential testing literature, the stopping boundaries $b_{1},\ldots ,b_{K}$ are chosen to take into account multiple comparisons and ensure that the resulting procedure preserves the desired overall type 1 error.^1^, ^2^, ^3^, ^9^ Standard methods^2^, ^3^, ^9^ for deriving stopping boundaries are based on the premise that the sequentially‐computed test statistics $T(t_{1}),\ldots ,T(t_{K})$ have the so‐called independent increments structure.^8^, ^16^ In the Supplemental Material, we sketch an argument demonstrating that, with the optimal choices of f(X) and $h\{u,X,A,\bar{L}(u)\}$ given in (19), the proposed test statistics, properly normalized, have the independent increments structure. Owing to this property, the practical strategies for interim monitoring presented in Section 4 can be implemented using standard software for computation of stopping boundaries; in the simulations in Section 5, we use the R package ldbounds.^17^

## 4 PRACTICAL IMPLEMENTATION AND INTERIM MONITORING STRATEGIES

### 4.1 Treatment effect estimation

Generalizing the approach in Tsiatis et al^10^ in the special case of a proportional odds model (2), we propose estimation of β at an interim analysis at time t using an AIPWCC estimator solving (17), which can be obtained via a two‐step algorithm.

Assume that the treatment effect β of interest is defined within a model for which, given full data at the end of the study, the estimator for β would be obtained jointly with that for α by solving an estimating equation of the form in (4) for a particular estimating function ℳ(Y,A,X;α,β). Because the optimal choices $f^{opt}(X)$ and $h^{opt}\{u,X,A,\bar{L}(u)\}$ in (19) are not known, we approximate them by linear combinations of basis functions. Letting $f_{0}(X),f_{1}(X),\ldots ,f_{M}(X)$ be functions of X specified by the analyst, with $f_{0}(X)\equiv 1$, approximate $f^{opt}(X)$ by

(22) $$\overset{M}{\underset{m=0}{\sum }}\psi_{m}f_{m}(X).$$

Similarly, specify basis functions of $\left\{u,X,\bar{L}(u)\right\}$, $h_{1}\{u,X,\bar{L}(u)\},\ldots ,h_{L}\{u,X,\bar{L}(u)\}$, and approximate $h^{opt}\{u,X,a,\bar{L}(u)\}$ by

(23) $$\overset{L}{\underset{\ell =1}{\sum }}\phi_{a,\ell }h_{\ell }\{u,X,\bar{L}(u)\},a=0,1.$$

With suitably chosen basis functions, experience in other contexts^6^, ^10^, ^15^ suggests that this approach can lead to AIPWCC estimators that achieve substantial efficiency gains over IPWCC estimators.

The AIPWCC estimator for β obtained by substituting (22) and (23) in (17) has influence function (16) with these same substitutions. Because from semiparametric theory the variance of the estimator depends on the variance of the influence function, as at the end of Section 2.1, we find the coefficients $\psi_{m}$, m=1,…,M, and $\phi_{a,\ell }$, ℓ=1,…,L, a=0,1, that minimize this variance, which, from the form of (16), is a least squares problem, as detailed below.

With these considerations, the two‐step algorithm is as follows. At an interim analysis at time t: *Step 1*. Estimate α and β by solving jointly in α and β

$$\overset{n}{\underset{i=1}{\sum }}I(E_{i}\leq t)\left[\frac{\Delta_{i}(t)ℳ(Y_{i},A_{i},X_{i};\alpha ,\beta )}{{\hat{𝒦}}_{t}\{U_{i}(t),A_{i}\}}\right]=0,$$

to obtain $\hat{\alpha }(t)$ and ${\hat{\beta }}^{init}(t)$; ${\hat{\beta }}^{init}(t)$ is an IPWCC estimator solving (18). Then obtain an estimator $\hat{𝒢}\{\hat{\alpha }(t),{\hat{\beta }}^{init}(t)\}$ for 𝒢(α,β). If the expectation in (7) is analytically tractable, $\hat{𝒢}\{\hat{\alpha }(t),{\hat{\beta }}^{init}(t)\}$ is the last row of (7) with $\hat{\alpha }(t)$ and ${\hat{\beta }}^{init}(t)$ substituted for α and β; if not, take the estimator $\hat{𝒢}\{\hat{\alpha }(t),{\hat{\beta }}^{init}(t)\}$ to be the last row of

$$-{\left[n{\left(t\right)}^{-1}\overset{n}{\underset{i=1}{\sum }}I(E_{i}\leq t)\frac{\Delta_{i}(t)}{{\hat{𝒦}}_{t}\{U_{i}(t),A_{i}\}}{\left.\frac{\partial ℳ(Y_{i},A_{i},X_{i};\alpha ,\beta )}{\partial \alpha^{T}\partial \beta }\right|}_{\alpha =\hat{\alpha }(t),\beta ={\hat{\beta }}^{init}(t)}\right]}^{-1}.$$

For each subject i for whom $E_{i}\leq t$, based on (8), construct

$$m\{Y_{i},A_{i},X_{i};\hat{\alpha }(t),{\hat{\beta }}^{init}(t)\}=\hat{𝒢}\{\hat{\alpha }(t),{\hat{\beta }}^{init}(t)\}ℳ\{Y_{i},A_{i},X_{i};\hat{\alpha }(t),{\hat{\beta }}^{init}(t)\}.$$

*Step 2*. Estimate the coefficients $\psi_{m}$, m=1,…,M, and $\phi_{a,\ell }$, ℓ=1,…,L, a=0,1, in the approximations (22) and (23) by “least squares,” as suggested above. Namely, for each subject i for whom $E_{i}\leq t$, define the “dependent variable”

$${\hat{Y}}_{i}(t)=\frac{\Delta_{i}(t)m\{Y_{i},A_{i},X_{i};\hat{\alpha }(t),{\hat{\beta }}^{init}(t)\}}{{\hat{𝒦}}_{t}\{U_{i}(t),A_{i}\}}+\int_{0}^{t}\frac{d{\hat{M}}_{ci}^{\left(t\right)}(u,A_{i})\hat{\mu }\{m,u,A_{i};\hat{\alpha }(t),{\hat{\beta }}^{init}(t)\}}{{\hat{𝒦}}_{t}(u,A_{i})},$$

where, in the integrand in the second term of the above expression, for $A_{i}=a$, a=0,1,

$$\begin{array}{llll} \frac{\hat{\mu }\{m,u,a;\hat{\alpha }(t),{\hat{\beta }}^{init}(t)\}}{{\hat{𝒦}}_{t}(u,a)} & ={\left\{\overset{n}{\underset{k=1}{\sum }}I(E_{k}\leq t){𝒴}_{k}^{\left(t\right)}(u)I(A_{k}=a)\right\}}^{-1}\; & & \\ & \;\times \overset{n}{\underset{k=1}{\sum }}\left\{\frac{I(E_{k}\leq t)\Delta_{k}(t)m\{Y_{k},A_{k},X_{k};\hat{\alpha }(t),{\hat{\beta }}^{init}(t)\}}{{\hat{𝒦}}_{t}\{U_{k}(t),a\}}{𝒴}_{k}^{\left(t\right)}(u)I(A_{k}=a)\right\}.\; & & \end{array}$$

Likewise, for each of these subjects and suitably chosen basis functions as discussed above, define the M+1+2L “covariates”

$$(A_{i}-{\hat{\pi }}_{t})f_{m}(X_{i}),\;m=0,1,\ldots ,M;$$

$$I(A_{i}=0)\int_{0}^{t}d{\hat{M}}_{ci}^{\left(t\right)}(u,0)\left[h_{\ell }\{u,X_{i},{\bar{L}}_{i}(u)\}-\hat{\mu }(h_{\ell },u,0)\right],\;\ell =1,\ldots ,L,$$

$$I(A_{i}=1)\int_{0}^{t}d{\hat{M}}_{ci}^{\left(t\right)}(u,1)\left[h_{\ell }\{u,X_{i},{\bar{L}}_{i}(u)\}-\hat{\mu }(h_{\ell },u,1)\right],\;\ell =1,\ldots ,L,$$

where, for a=0,1

$$\hat{\mu }(h_{\ell },u,a)={\left\{\overset{n}{\underset{k=1}{\sum }}I(E_{k}\leq t){𝒴}_{k}^{\left(t\right)}(u)I(A_{k}=a)\right\}}^{-1}\overset{n}{\underset{k=1}{\sum }}h_{\ell }\{u,X_{k},{\bar{L}}_{k}(u)\}{𝒴}_{k}^{\left(t\right)}(u)I(A_{k}=a).$$

Then obtain estimators ${\hat{\psi }}_{m}$, m=0,1,…,M, and ${\hat{\phi }}_{a,\ell }$, ℓ=1,…,L, a=0,1, by linear regression of ${\hat{Y}}_{i}(t)$ on the above covariates. Based on this regression, obtain “predicted values” for each subject i for whom $E_{i}\leq t$ as

$$\begin{array}{ll} Pred_{i}=(A_{i}-{\hat{\pi }}_{t})\overset{M}{\underset{m=0}{\sum }}{\hat{\psi }}_{m}f_{m}(X_{i})+\int_{0}^{t}d{\hat{M}}_{ci}^{\left(t\right)}(u,A_{i})\overset{L}{\underset{\ell =1}{\sum }}{\hat{\phi }}_{A_{i},\ell }\left[h_{\ell }\{u,X_{i},{\bar{L}}_{i}(u)\}-\hat{\mu }(h_{\ell },u,A_{i})\right]. & \end{array}$$

The estimator for β is then obtained as the one‐step update

(24) $$\hat{\beta }(t)={\hat{\beta }}^{init}(t)-n{\left(t\right)}^{-1}\overset{n}{\underset{i=1}{\sum }}I(E_{i}\leq t)Pred_{i},$$

and an approximate SE for $\hat{\beta }(t)$ is given by

(25) $$SE\{\hat{\beta }(t)\}=n{\left(t\right)}^{-1}{\left[\overset{n}{\underset{i=1}{\sum }}I(E_{i}\leq t){\left\{{\hat{Y}}_{i}(t)-Pred_{i}\right\}}^{2}\right]}^{1/2}.$$

By an argument similar to that in the Supplementary Material of Tsiatis et al,^10^ the estimator (24) is asymptotically equivalent to an AIPWCC estimator solving (17).

In some settings, scant time‐dependent covariate information may be available. Here, a special case of the general AIPWCC formulation that still attempts to gain efficiency from only baseline covariates X is to solve an estimating equation of the form in (17) but with f(X) as in (19) and $h\{u,X,A,\bar{L}(u)\}=0$. Implementation is as above, but with the “dependent variable” in Step 2 regressed only on the M+1 “covariates” $\left(A_{i}-{\hat{\pi }}_{t})f_{m}(X_{i}\right)$, m=0,1,…,M, to obtain estimators ${\hat{\psi }}_{m}$, m=0,1,…,M, and by redefining $Pred_{i}=(A_{i}-{\hat{\pi }}_{t})\sum_{m=0}^{M}{\hat{\psi }}_{m}f_{m}(X_{i})$ in the one‐step update (24) and its associated standard error (25). For definiteness, we refer to the resulting estimator as “AIPW1” and that incorporating time‐dependent covariates above as “AIPW2.”

### 4.2 Interim analysis

There is a vast literature on early stopping of clinical trials using group sequential and other methods; such methods are readily applied if the independent increments property holds. We now discuss information‐based and fixed‐sample size monitoring strategies for using these approaches with the proposed treatment effect estimators, for which, as argued in the Supplemental Material and demonstrated empirically in Section 5, the independent increments property holds exactly or approximately.

In the general information‐based monitoring approach,^1^ monitoring and group sequential tests are based on the proportion of the total information to be gained from the completed trial available at interim analysis times t, where in the present context information is approximated at time t using the large‐sample approximate SE of the relevant estimator $\hat{\beta }(t)$, $SE\{\hat{\beta }(t)\}$. If a group sequential test is desired with type 1 error α for testing $H_{0}:\beta_{0}=0$ and power (1−γ) against a clinically meaningful alternative value $\beta_{0}=\beta_{A}$, say, then the maximum information MI required to achieve this objective at the final analysis with a two‐sided test is

$$MI={\left(\frac{z_{\alpha /2}+z_{\gamma }}{\beta_{A}}\right)}^{2}IF,$$

where $z_{\delta }$ is the (1−δ) quantile of the standard normal distribution, and IF is an inflation factor to account for the loss of power that results due to repeated testing relative to doing a single final analysis. For example, the inflation factor associated with using O'Brien‐Fleming stopping boundaries^3^ is modest, equal to about 1.03; see Tsiatis.^1^ Information at an interim analysis at time t is approximated as

$$\mathrm{Inf}(t)={\left[SE\{\hat{\beta }(t)\}\right]}^{-2}.$$

Thus, the proportion of information at interim analysis time t is approximated as

(26) $$p(t)=\frac{\mathrm{Inf}(t)}{MI}.$$

Given the proportion of information (26) together with, for example, the Lan‐DeMets spending function,^9^ standard software can be used to obtain stopping boundaries such that the resulting group sequential testing procedure has the desired operating characteristics.

Typically, in determining the overall sample size for a clinical trial to achieve the desired power to detect a meaningful difference at a given level of significance, the analyst must make assumptions on the values of nuisance parameters. If these assumptions are not correct, the trial could be underpowered. An oft‐cited advantage of information‐based monitoring is that interim analyses would continue until the information Inf(t) achieves the maximum information MI, guaranteeing the desired operating characteristics regardless of the values of nuisance parameters. However, if the assumptions leading to the target sample size are not correct, the information available at the time this planned sample size is reached and all participants have the outcome ascertained may be less than MI. If evidence emerges during the trial that MI is unlikely to be achieved, the sample size might be reestimated and increased so that the full information threshold MI is met.

In many trials, however, resource constraints or other factors may make exceeding the originally planned sample size impossible, thus rendering principled information‐based monitoring infeasible. If a fixed, maximum sample size $n_{\max}$, say, is planned and inalterable, then the proportion of information available at an interim analysis at any time t on which stopping boundaries can be determined must instead be based on $n_{\max}$. In terms of the data (11) available at an interim analysis at t, as before, $n(t)=\sum_{i=1}^{n}I(E_{i}\leq t)$ is the number of subjects who have enrolled by time t; of these subjects, $n_{A}(t)=\sum_{i=1}^{n}I\{E_{i}\leq t,C_{i}(t)\geq {𝒯}_{F}\}$ is the number who have been enrolled for the maximum follow‐up period and thus have the outcome ascertained with certainty, and it is likely that $n_{A}(t)<n(t)$. In general, a typical interim analysis at t for fixed $n_{\max}$ would be based only on the data from these $n_{A}(t)$ subjects, and, accordingly, the proportion of information available at t would be $p(t)=n_{A}(t)/n_{\max}$, with p(t)=1 at the final analysis. However, here, the proposed IPWCC and AIPWCC estimators allow censoring due to the time lag in ascertaining the outcome to be taken into account and incorporation of covariates to increase efficiency, so make use of additional information in (11) beyond that available on just the $n_{A}(t)$ subjects for whom the outcome has been ascertained by time t. Thus, if monitoring is based on test statistics constructed from these estimators, the proportion of information available at t should be between $n_{A}(t)/n_{\max}$ and $n(t)/n_{\max}$.

With these considerations, for fixed‐sample size monitoring, we propose characterizing the proportion of information available at an interim analysis at time t in terms of what we refer to as the effective sample size $n_{ESS}(t)$, say, at t. Intuitively, we define $n_{ESS}(t)$ to be the number of participants, had they been enrolled for the maximum follow‐up period ${𝒯}_{F}$ and had their outcome ascertained with certainty, that would be required to lead to an estimator for β based only on data from such subjects with the same precision as that achieved by an IPWCC or AIPWCC estimator for β based on all of the available data at t. The proportion of information at t would then be $n_{ESS}(t)/n_{\max}$.

To define effective sample size formally, with $n^{*}$ subjects for whom the outcome has been fully ascertained, indexed by $j=1,\ldots ,n^{*}$, consider the estimator ${\hat{\beta }}^{*}$ obtained by solving in β the full data estimating Equation (9) based on these $n^{*}$ subjects,

$$\overset{n^{*}}{\underset{j=1}{\sum }}m(Y_{j},A_{j},X_{j};\hat{\alpha },\beta )=0,$$

for some consistent estimator $\hat{\alpha }$. Then from the semiparametric theory, ${\hat{\beta }}^{*}$ has SE approximately equal to the square root of $\mathrm{var}\{m(Y,A,X;\alpha_{0},\beta_{0})\}/n^{*}.$ The effective sample size at an interim analysis at time t for the IPWCC estimator $\hat{\beta }(t)$ calculated using the available data (11) at t is the value $n^{*}$ such that $\mathrm{var}\{m(Y,A,X;\alpha_{0},\beta_{0})\}/n^{*}=SE{\left\{\hat{\beta }(t)\right\}}^{2}$. Accordingly, define the effective sample size when monitoring is based on the IPWCC estimator as

(27) $$n_{ESS}(t)=\frac{\mathrm{var}\{m(Y,A,X;\alpha_{0},\beta_{0})}{SE{\left\{\hat{\beta }(t)\right\}}^{2}}.$$

Because $\mathrm{var}\{m(Y,A,X;\alpha_{0},\beta_{0})\}$ is not known, in practice we must estimate it based on the available data, which can be accomplished via the estimator

(28) $$\hat{\mathrm{var}}\{m(Y,A,X;\alpha_{0},\beta_{0})\}=n{\left(t\right)}^{-1}\overset{n}{\underset{i=1}{\sum }}I(E_{i}\leq t)\frac{\Delta_{i}(t)m{\left\{Y_{i},A_{i},X_{i};\hat{\alpha }(t),\hat{\beta }(t)\right\}}^{2}}{{\hat{𝒦}}_{t}\{U_{i}(t),A_{i}\}}.$$

Thus, in practice, we obtain the approximate effective sample size as

(29) $$n_{ESS}(t)=\frac{\hat{\mathrm{var}}\{m(Y,A,X;\alpha_{0},\beta_{0})\}}{SE{\left\{\hat{\beta }(t)\right\}}^{2}}.$$

The effective sample size for an AIPWCC estimator $\hat{\beta }(t)$ (either AIPW1 or AIPW2) calculated using the available data at t via the two‐step algorithm is defined similarly, but with the full data influence function $m(Y,A,X;\alpha_{0},\beta_{0})$ in (27) replaced by the influence function $m(Y,A,X;\alpha_{0},\beta_{0})-(A-\pi )f^{opt}(X)$ as in (21). An estimator for $\mathrm{var}\{m(Y,A,X;\alpha_{0},\beta_{0})-(A-\pi )f^{opt}(X)\}$ based on the available data is given by

(30) $$\hat{\mathrm{var}}\{m(Y,A,X;\alpha_{0},\beta_{0})\}=n{\left(t\right)}^{-1}\overset{n}{\underset{i=1}{\sum }}I(E_{i}\leq t)\frac{\Delta_{i}(t){\left[m\{Y_{i},A_{i},X_{i};\hat{\alpha }(t),\hat{\beta }(t)\}-Pred_{i}^{*}\right]}^{2}}{{\hat{𝒦}}_{t}\{U_{i}(t),A_{i}\}},\;Pred_{i}^{*}=(A_{i}-{\hat{\pi }}_{t})\overset{M}{\underset{m=0}{\sum }}{\hat{\psi }}_{m}f_{m}(X_{i}),$$

where now the “predicted values” $Pred_{i}^{*}$ are obtained by a weighted least squares regression with “dependent variable” $m\{Y,A,X;\hat{\alpha }(t),\hat{\beta }(t)\}$, “covariates” $\left(A_{i}-{\hat{\pi }}_{t})f_{m}(X_{i}\right)$, m=0,1,…,M, and “weights” $\Delta_{i}(t)/{\hat{𝒦}}_{t}\{U_{i}(t),A_{i}\}$. Thus, for $\hat{\beta }(t)$ the AIPW1 or AIPW2 estimator, $n_{ESS}(t)$ is defined as in (29) with the numerator given by (30).

With the appropriate definition of $n_{ESS}(t)$, we approximate the corresponding proportion of information available at t with interim analyses based on an IPWCC or AIPWCCC estimator as

(31) $$p(t)=\frac{n_{ESS}(t)}{n_{\max}}.$$

From (28) and (30), p(t)=1 at the final analysis. As for information‐based monitoring, given the proportion of information (31), one can use standard software with, for example, the Lan‐DeMets spending function^9^ to obtain stopping boundaries.

In the simulation studies in the next section, we study the methods under fixed‐sample monitoring, as in our experience this approach is most common in practice. Moreover, while performance of information‐based monitoring with statistics that possess the independent increments property has been well‐studied,^8^, ^18^ because our approach to characterizing proportion of information in fixed‐sample monitoring based on the proposed effective sample size measure is new, evaluation of its performance is required.

## 5 SIMULATION STUDIES

We present results from several simulation studies, each involving 10 000 Monte Carlo replications. For each simulation scenario, we considered a uniform enrollment process during the calendar time interval $\left[0,E_{\max}\right]$ with a maximum, fixed sample size $n_{\max}$ and maximum follow up time ${𝒯}_{F}$, and fixed‐sample size monitoring with interim analyses planned at calendar times $t_{1}<\cdots <t_{K}$ and a final analysis at time $t_{end}=E_{\max}+{𝒯}_{F}=t_{K+1}>t_{K}$, for a total of K+1 possible analyses. For simplicity, in each scenario, we took $n_{\max}$ to be the sample size required to achieve roughly 80% or 90% power for a single analysis at $t_{end}$ and did not include an inflation factor.^1^ At each of the K+1 analysis times, we estimated the relevant treatment effect parameter β four ways:

(i) using the estimator ${\hat{\beta }}_{{𝒯}_{F}}(t_{j})$ obtained by carrying out the full data analysis based only on subjects enrolled for at least the maximum follow‐up period ${𝒯}_{F}$, so using subjects i for whom $I\{E_{i}\leq t,C_{i}(t)\geq {𝒯}_{F}\}=1$;
(ii) using the IPWCC estimator ${\hat{\beta }}_{IPW}(t_{j})={\hat{\beta }}^{init}(t_{j})$ based on the available data (11), obtained at Step 1 of the two‐step algorithm;
(iii) using the AIPWCC estimator ${\hat{\beta }}_{AIPW1}(t_{j})$ based on the available data (11), obtained at Step 2 of the two‐step algorithm using only baseline covariates X to gain efficiency, as at the end of Section 4.1;
(iv) using the AIPWCC estimator ${\hat{\beta }}_{AIPW2}(t_{j})$ based on the available data (11), obtained at Step 2 of the algorithm using both baseline and time‐dependent covariates X and $\bar{L}(u)$.

At the final analysis at time $t_{end}$ at which the outcome has been ascertained on all $n_{\max}$ subjects, ${\hat{\beta }}_{{𝒯}_{F}}(t_{end})$ and ${\hat{\beta }}_{IPW}(t_{end})$ yield (versions of) the intended full data analysis; and ${\hat{\beta }}_{AIPW1}(t_{end})$ and ${\hat{\beta }}_{AIPW2}(t_{end})$ are identical and yield the covariate‐adjusted analysis exploiting baseline covariate information discussed at the end of Section 3.1. In all scenarios, for the null hypothesis $H_{0}:\beta_{0}=0$ and one‐sided alternative hypotheses and level of significance α=0.025, we used the R package ldbounds^17^ with a Lan‐DeMets spending function to compute both O'Brien‐Fleming^3^ and Pocock^2^ stopping boundaries at each analysis time $t_{j}$, j=1,…,K+1. For ${\hat{\beta }}_{{𝒯}_{F}}(t_{j})$, this calculation was based on the proportion of information $p(t_{j})=n_{A}(t_{j})/n_{\max}$; for each of the IPWCC and AIPWCC estimators ${\hat{\beta }}_{IPW}(t_{j})$, ${\hat{\beta }}_{AIPW1}(t_{j})$, and ${\hat{\beta }}_{AIPW2}(t_{j})$, the stopping boundaries were obtained using the approximate proportion of information (31) based on the relevant approximate effective sample size $n_{ESS}(t_{j})$ given in (29).

For each scenario, we present the following results from two simulation studies, one under $H_{0}$, so with data generated with $\beta_{0}=0$, and one under an alternative of interest $\beta_{0}=\beta_{A}$:

(i) for each estimator, Monte Carlo estimates of $\text{cov}\{\hat{\beta }(s),\hat{\beta }(t)\}$, s<t, and $\mathrm{var}\{\hat{\beta }(t)\}$, $s,t\in \{t_{1},\ldots ,t_{K},t_{end}\}$; if the independent increments property holds, $\text{cov}\{\hat{\beta }(s),\hat{\beta }(t)\}=\mathrm{var}\{\hat{\beta }(t)\}$, s<t;
(ii) for each estimator at each $t\in \{t_{1},\ldots ,t_{K},t_{end}\}$, Monte Carlo mean and SD of $\hat{\beta }(t)$, Monte Carlo mean of $SE\{\hat{\beta }(t)\}$, and Monte Carlo mean square error (MSE) for ${\hat{\beta }}_{{𝒯}_{F}}(t)$ divided by that for $\hat{\beta }(t)$;
(iii) for each estimator and stopping boundary, the Monte Carlo proportion of data sets for which $H_{0}$ was rejected, Monte Carlo estimate of expected sample size, and Monte Carlo estimate of expected stopping time.

The first two simulation scenarios, demonstrating the methods for an ordinal categorical outcome and a binary outcome, respectively, are based on the TESICO study with ${𝒯}_{F}=90$ days, using the generative models adopted by Tsiatis et al,^10^ with $n_{\max}=602$, $E_{\max}=240$ days, and K=4 interim analyses planned at calendar times $\left(t_{1},\ldots ,t_{4})=(150,195,240,285\right)$ days, with the final analysis at $t_{end}=330$ days. For each simulated subject, A was generated as Bernoulli with pr(A=1)=π=0.5, where a=0 (1) corresponds to placebo (active agent). To produce data for which the proportional odds model (2) holds, we generated Υ∼U(0,1) and set $\Gamma =(1-A)\Upsilon +A\Upsilon (1/\text{OR})/\{1-\Upsilon +\Upsilon (1/e^{\beta })\}$, where as in (2) β is the log odds ratio, so that the distribution of Γ given A=1 satisfies logit{pr(Γ≤u|A=1)}=logit{pr(Γ≤u|A=0)}+β. For Y an ordinal outcome, we took pr(Y=j|A=0)=0.12,0.23,0.17,010,0.05,0.33 for j=1,…,6 as in Table 1 of Tsiatis et al^10^ and thus generated Y according to in which interval Γ fell as determined by the cutpoints [0.00,0.12,0.35,0.52,0.62,0.67,1.00]. Then if Γ<0.52, so Y=1,2, or 3, we took the time in hospital to be $H={𝒯}_{F}\Gamma /0.52$ and the number of days at home and off oxygen as ${𝒯}_{F}-H$, and $T={𝒯}_{F}$. If 0.52≤Γ<0.62 or 0.62≤Γ<0.67, corresponding to Y=4 or 5, again $T={𝒯}_{F}$; if Γ≥0.67, corresponding to death, time of death $T=(1-A)T_{0}+AT_{1}$, where $T_{0}∼U(0,30)$ if A=0 and $T_{1}∼U(20,50)$ if A=1. A baseline covariate was generated as X∼𝒩{1.5(Υ−0.5),1}, so that X is independent of A, correlated with Y, and does not affect the proportional odds model. Two time dependent covariates were generated as $L_{1}(u)=I(𝒲<u)$, where $𝒲=HI(\Gamma <0.52)+{𝒯}_{F}I(\Gamma \geq 0.52)$, so that $L_{1}(u)=1$ if the subject was still in the hospital at time u; and $L_{2}(u)=({𝒯}_{F}-𝒲)L_{1}(u)$, the number of days the subject was expected to be out of the hospital at day ${𝒯}_{F}$, and $L(u)=\{L_{1}(u),L_{2}(u)\}$. For a scenario with binary outcome, we generated the data according to the foregoing scheme, except that we defined Y=1, corresponding to death, if Γ≥0.67, and Y=0 otherwise.

For the first scenario with ordinal categorical outcome, we generated data as above under the null hypothesis, so with $\beta =\beta_{0}=0$, and $\beta =\beta_{A}=\log(1.5)$, corresponding to the alternative for which TESICO was powered (80% at the final analysis with n=602),^10^ and $H_{A}:\beta_{0}>0$. Here, ${\hat{\beta }}_{{𝒯}_{F}}(t)$ is the ML estimator for β in (2) obtained using the R function polr in the MASS package.^19^ Following Tsiatis et al,^10^ to simplify implementation, we constructed ${\hat{\beta }}_{IPW}(t)$, ${\hat{\beta }}_{AIPW1}(t)$, and ${\hat{\beta }}_{AIPW2}(t)$ using the estimating function (6) with $𝒟(A)=D^{T}(A;\alpha ,\beta )V_{ind}^{-1}(A;\alpha ,\beta )$, where $V_{ind}(A;\alpha ,\beta )$ is chosen according to the “working independence” assumption, so that with full data at the final analysis, ${\hat{\beta }}_{{𝒯}_{F}}(t_{end})$ and ${\hat{\beta }}_{IPW}(t_{end})$ are not identical. As shown by Tsiatis et al^10^ and borne out in the simulations below, the efficiency loss for ${\hat{\beta }}_{IPW}(t_{end})$ relative to ${\hat{\beta }}_{{𝒯}_{F}}(t_{end})$ is negligible.

Under (a) the null hypothesis and (b) the alternative, the Monte Carlo sample covariance matrices of the 10 000 estimates $\left\{{\hat{\beta }}_{AIPW2}(t_{1}),\ldots ,{\hat{\beta }}_{AIPW2}(t_{4}),{\hat{\beta }}_{AIPW2}(t_{end})\right\}$ are

(32) $$\text{(a)}\;\left(\begin{array}{ccccc} 0.041 & 0.027 & 0.022 & 0.019 & 0.019\\ 0.027 & 0.028 & 0.021 & 0.019 & 0.018\\ 0.022 & 0.021 & 0.022 & 0.019 & 0.019\\ 0.019 & 0.019 & 0.019 & 0.019 & 0.018\\ 0.019 & 0.018 & 0.019 & 0.018 & 0.018 \end{array}\right),\;\text{(b)}\;\left(\begin{array}{ccccc} 0.042 & 0.027 & 0.022 & 0.019 & 0.019\\ 0.027 & 0.029 & 0.022 & 0.019 & 0.019\\ 0.022 & 0.022 & 0.022 & 0.019 & 0.019\\ 0.019 & 0.019 & 0.019 & 0.019 & 0.019\\ 0.019 & 0.019 & 0.019 & 0.019 & 0.019 \end{array}\right).$$

In (32), it is evident that $\text{cov}\{\hat{\beta }(s),\hat{\beta }(t)\}\approx \mathrm{var}\{\hat{\beta }(t)\}$, s<t, demonstrating that the independent increments property holds approximately for this estimator. Analogous results for the other three estimators are given in the Supplemental Material, showing that the independent increments property holds approximately for all.

Under the null hypothesis and the alternative, Table 1 presents Monte Carlo mean and SD, the Monte Carlo average of standard errors $SE\{\hat{\beta }(t)\}$, and MSE ratio defined above as the Monte Carlo MSE for ${\hat{\beta }}_{{𝒯}_{F}}(t)$ divided by that for the indicated estimator. Thus, the MSE ratio reflects efficiency of the indicated estimator relative to the usual ML estimator using data only on subjects enrolled for the maximum follow‐up period ${𝒯}_{F}$. From the table, all estimators are consistent, with SEs that track the Monte Carlo SDs, under both hypotheses. The efficiency gains over ${\hat{\beta }}_{{𝒯}_{F}}(t)$ achieved at interim analyses by using any of ${\hat{\beta }}_{IPW}(t)$, ${\hat{\beta }}_{AIPW1}(t)$, and ${\hat{\beta }}_{AIPW2}(t)$ are substantial. The IPWCC estimator achieves gains solely through accounting for censoring; the AIPWCC estimators improve on these gains by additionally incorporating covariates. Notably, ${\hat{\beta }}_{AIPW2}(t)$ yields a 2‐fold gain at the initial interim analysis. For all three estimators, the efficiency gains are most pronounced at the early interim analyses where censoring is the most substantial and diminish as censoring decreases as the trial progresses. At the final analysis, ${\hat{\beta }}_{{𝒯}_{F}}(t_{end})$ and ${\hat{\beta }}_{IPW}(t_{end})$ show very similar performance, with ${\hat{\beta }}_{IPW}(t_{end})$ exhibiting minimal relative loss of efficiency, as noted above. As expected, ${\hat{\beta }}_{AIPW1}(t_{end})$ and ${\hat{\beta }}_{AIPW2}(t_{end})$ are identical and, due to the incorporation of adjustment for baseline covariates, result a 16%‐17% gain in efficiency over the usual final analysis.

**TABLE 1:** For Scenario 1 with ordered categorical outcome, performance of estimators for β under (a) the null hypothesis β=0 and (b) the alternative β=log(1.5)=0.405 at each interim analysis time $\left(t_{1},\ldots ,t_{4})=(150,195,240,285\right)$ days and at the final analysis at $t_{end}=330$ days

	MC mean	MC SD	Ave MC SE	MSE ratio	MC mean	MC SD	Ave MC SE	MSE ratio
	(a) Null hypothesis							
	${\hat{\beta }}_{{𝒯}_{F}}(t)$				${\hat{\beta }}_{IPW}(t)$			
$t_{1}$	−0.002	0.294	0.294	1.000	−0.004	0.232	0.232	1.603
$t_{2}$	−0.002	0.221	0.221	1.000	−0.002	0.189	0.189	1.330
$t_{3}$	−0.003	0.184	0.185	1.000	−0.002	0.166	0.164	1.239
$t_{4}$	−0.001	0.162	0.162	1.000	−0.001	0.152	0.151	1.139
$t_{end}$	0.000	0.146	0.146	1.000	0.000	0.147	0.146	0.991
	${\hat{\beta }}_{AIPW1}(t)$				${\hat{\beta }}_{AIPW2}(t)$			
$t_{1}$	−0.004	0.221	0.221	1.775	−0.005	0.203	0.198	2.095
$t_{2}$	−0.002	0.178	0.178	1.534	−0.002	0.168	0.165	1.717
$t_{3}$	−0.002	0.156	0.154	1.399	−0.002	0.149	0.145	1.542
$t_{4}$	−0.001	0.141	0 .140	1.327	−0.001	0.138	0.136	1.380
$t_{end}$	0.000	0.135	0.135	1.169	0.000	0.135	0.135	1.169

	(b) Alternative hypothesis							
	${\hat{\beta }}_{{𝒯}_{F}}(t)$				${\hat{\beta }}_{IPW}(t)$			
$t_{1}$	0.408	0.294	0.294	1.000	0.406	0.235	0.235	1.566
$t_{2}$	0.406	0.220	0.221	1.000	0.406	0.191	0.191	1.336
$t_{3}$	0.404	0.185	0.185	1.000	0.405	0.167	0.165	1.221
$t_{4}$	0.406	0.163	0.162	1.000	0.406	0.153	0.152	1.131
$t_{end}$	0.406	0.147	0.146	1.000	0.406	0.148	0.147	0.985

	${\hat{\beta }}_{AIPW1}(t)$				${\hat{\beta }}_{AIPW2}(t)$			
$t_{1}$	0.405	0.224	0.224	1.733	0.406	0.204	0.200	2.078
$t_{2}$	0.406	0.180	0.180	1.508	0.408	0.169	0.167	1.702
$t_{3}$	0.405	0.158	0.155	1.378	0.408	0.150	0.147	1.523
$t_{4}$	0.406	0.142	0.141	1.314	0.407	0.139	0.137	1.373
$t_{end}$	0.406	0.137	0.136	1.159	0.406	0.137	0.136	1.159

*Note*: MC mean is the mean of 10 000 Monte Carlo estimates; MC SD is the Monte Carlo SD, Ave MC SE is the mean of Monte Carlo SEs, and MSE ratio is the ratio of Monte Carlo mean square error for ${\hat{\beta }}_{{𝒯}_{F}}(t)$ divided by that for the indicated estimator.

Table 2 presents interim monitoring results using each estimator with both O'Brien‐Fleming and Pocock stopping boundaries under the null hypothesis and under the alternative β=log(1.5). Under the null, the nominal level α=0.025 is achieved for all estimators. Under the alternative, power for ${\hat{\beta }}_{{𝒯}_{F}}(t)$ is slightly shy of the desired 80%, as expected with no inflation factor; by comparison, the AIPWCC estimators yield improved power due to inclusion of covariate information. Under the alternative and both types of boundaries, basing interim analyses on ${\hat{\beta }}_{IPW}(t)$, ${\hat{\beta }}_{AIPW1}(t)$, and ${\hat{\beta }}_{AIPW2}(t)$ results in impressive reductions in expected sample size and expected stopping time relative to ${\hat{\beta }}_{{𝒯}_{F}}(t)$, with the gains especially impressive for ${\hat{\beta }}_{AIPW2}(t)$.

**TABLE 2:** For Scenario 1 with ordered categorical outcome, interim analysis performance using each estimator with O'Brien‐Fleming and Pocock stopping boundaries under (a) the null hypothesis β=0 and (b) the alternative β=log(1.5)=0.405, with maximum sample size $n_{\max}=602$ and $t_{end}=330$ days

	P(reject)	MC E(SS)	MC E(Stop)	P(reject)	MC E(SS)	MC E(Stop)
	(a) Null hypothesis					
	O'Brien‐Fleming			Pocock		
${\hat{\beta }}_{{𝒯}_{F}}(t)$	0.024	601.9 (2.7)	329.3 (7.5)	0.023	599.7 (21.4)	327.4 (19.5)
${\hat{\beta }}_{IPW}(t)$	0.024	601.6 (7.8)	328.4 (12.1)	0.024	598.8 (25.9)	326.7 (22.4)
${\hat{\beta }}_{AIPW1}(t)$	0.024	601.7 (6.7)	328.5 (11.3)	0.024	598.9 (25.5)	326.8 (22.0)
${\hat{\beta }}_{AIPW2}(t)$	0.024	601.2 (11.4)	327.9 (14.9)	0.027	598.0 (28.9)	326.1 (24.7)

	(b) Alternative hypothesis					
	O'Brien‐Fleming			Pocock		
${\hat{\beta }}_{{𝒯}_{F}}(t)$	0.784	592.7 (31.5)	284.2 (44.7)	0.710	548.1 (85.3)	260.5 (68.7)
${\hat{\beta }}_{IPW}(t)$	0.771	564.6 (62.5)	257.1 (55.9)	0.701	516.3 (100.3)	239.0 (74.7)
${\hat{\beta }}_{AIPW1}(t)$	0.836	562.7 (62.7)	251.5 (53.4)	0.774	508.3 (100.6)	230.1 (72.2)
${\hat{\beta }}_{AIPW2}(t)$	0.841	531.9 (81.7)	231.7 (58.0)	0.783	483.4 (103.5)	215.1 (72.3)

*Note*: P(reject) is the proportion of Monte Carlo data sets for which the null hypothesis was rejected; MC E(SS) is the Monte Carlo average of number of subjects enrolled at the time the stopping boundary was crossed (SD); and MC E(Stop) is the Monte Carlo average stopping time (days) (SD). The standard error for entries for P(reject) in (a) is ≈0.0016.

For the second scenario with binary outcome, we generated data as above under the null hypothesis and with the log odds ratio equal to 1.5, which implies a log relative risk (risk ratio) for death (Y=1) of $\beta =\beta_{0}=\beta_{A}=\log(0.247/0.33)=-0.290$ as in (3) and alternative hypothesis $H_{A}:\beta_{0}<0$. We took $n_{\max}=900$, which corresponds roughly to 90% power to detect this alternative. The Monte Carlo sample covariance matrices of the 10 000 estimates $\left\{\hat{\beta }(t_{1}),\ldots ,\hat{\beta }(t_{4}),\hat{\beta }(t_{end})\right\}$ for each of the four estimators under both the null and alternative settings are shown in the Supplemental Material and exhibit patterns analogous to those in (32), demonstrating that all estimators have approximately the independent increments property. Also shown in the Supplemental Material for each estimator at each analysis time are the Monte Carlo mean and SD, the Monte Carlo average of standard errors $SE\{\hat{\beta }(t)\}$, and MSE ratio defined above as the Monte Carlo MSE for the estimator divided by that for ${\hat{\beta }}_{{𝒯}_{F}}(t)$ under the null and alternative hypotheses. All estimators are consistent, and SEs are very close to the Monte Carlo SDs. Under both null and alternative hypotheses, the estimator ${\hat{\beta }}_{IPW}(t)$, which takes censoring at interim analysis times into account and as noted previously is identical to the ratio of treatment‐specific Kaplan‐Meier estimators often used in practice, achieves substantial efficiency gains over ${\hat{\beta }}_{{𝒯}_{F}}(t)$, with a 2‐fold increase at the first interim analysis and 24% at the last at $t_{4}=285$ days. These estimators are equivalent, as expected, at the final analysis. The AIPWCC estimators ${\hat{\beta }}_{AIPW1}(t)$ and ${\hat{\beta }}_{AIPW2}(t)$ achieve even greater gains. Here, ${\hat{\beta }}_{AIPW2}(t)$ does not offer improved performance over ${\hat{\beta }}_{AIPW1}(t)$; this behavior is not surprising, as the time‐dependent covariates $L(u)=\{L_{1}(u),L_{2}(u)\}$ reflecting length of hospital stay do not provide information on death. As expected, these estimators are identical at $t_{end}$ and offer 10%‐12% gains in efficiency over the standard analysis through adjustment for the baseline covariate.

Table 3 shows interim monitoring results using each estimator with O'Brien‐Fleming and Pocock stopping boundaries under the null hypothesis and under the alternative $\beta_{A}=-0.290$. Again, overall testing procedures achieve the nominal level. Power gains over ${\hat{\beta }}_{{𝒯}_{F}}(t)$ under the alternative are achieved using the AIPWCC estimators. As for the first scenario, basing interim analyses on ${\hat{\beta }}_{IPW}(t)$, ${\hat{\beta }}_{AIPW1}(t)$, and ${\hat{\beta }}_{AIPW2}(t)$ yields substantial reductions in expected sample size and stopping time over ${\hat{\beta }}_{{𝒯}_{F}}(t)$ under the alternative, especially for the AIPWCC estimators.

**TABLE 3:** For Scenario 2 with binary outcome, interim analysis performance using each estimator with O'Brien‐Fleming and Pocock stopping boundaries under (a) the null hypothesis β=0 and (b) the alternative β=log(0.247/0.33)=−0.290, with maximum sample size $n_{\max}=900$ and $t_{end}=330$ days

	P(reject)	MC E(SS)	MC E(Stop)	P(reject)	MC E(SS)	MC E(Stop)
	(a) Null hypothesis					
	O'Brien‐Fleming			Pocock		
${\hat{\beta }}_{{𝒯}_{F}}(t)$	0.024	900.0 (2.3)	329.4 (6.5)	0.022	896.3 (33.6)	327.4 (19.8)
${\hat{\beta }}_{IPW}(t)$	0.024	898.8 (15.8)	328.0 (14.5)	0.023	894.3 (42.3)	326.5 (23.7)
${\hat{\beta }}_{AIPW1}(t)$	0.023	899.0 (14.0)	328.1 (13.8)	0.025	894.1 (42.8)	326.3 (24.3)
${\hat{\beta }}_{AIPW2}(t)$	0.024	899.0 (14.9)	328.0 (14.2)	0.026	894.1 (42.7)	326.2 (24.4)

	(b) Alternative hypothesis					
	O'Brien‐Fleming			Pocock		
${\hat{\beta }}_{{𝒯}_{F}}(t)$	0.770	887.5 (44.3)	285.9 (44.0)	0.690	827.2 (122.4)	264.4 (67.2)
${\hat{\beta }}_{IPW}(t)$	0.767	808.3 (121.4)	241.3 (61.3)	0.700	744.9 (157.6)	228.3 (76.7)
${\hat{\beta }}_{AIPW1}(t)$	0.806	801.8 (122.5)	236.1 (59.6)	0.746	733.2 (157.3)	221.4 (74.7)
${\hat{\beta }}_{AIPW2}(t)$	0.809	799.9 (123.4)	235.5 (59.7)	0.748	731.6 (157.3)	220.6 (74.7)

*Note*: Entries are as in Table 2. The standard error for entries for P(reject) in (a) is ≈0.0016.

The final simulation scenario involves a continuous outcome, with $n_{\max}=300$, $E_{\max}=156$ weeks, and ${𝒯}_{F}=52$ weeks, so that enrollment takes place over 3 years, with K=4 interim analyses planned at calendar times 104, 130, 156, 182 weeks and the final analysis at $t_{end}=208$ weeks. We generated treatment assignment A as Bernoulli with pr(A=1)=π=0.5, where a=0 (1) corresponds to placebo (active agent); and a categorical baseline covariate $X_{1}$ was generated with $\mathrm{pr}(X_{1}=j)=0.4,0.3,0.2,0.1$ for j=1,…,4. With $\left(s_{1},\ldots ,s_{5})=(0,4,12,24,52\right)$ weeks, σ=4.5, D a (2×2) matrix with vech(D)=(80,−0.5,0.08), and $\xi ={\left(\xi_{1},\xi_{2}\right)}^{T}$, we generated longitudinal measurements for each subject i according to the linear mixed effects model $Z_{ij}=65I(X_{1}=1)+60I(X_{1}=2)+55I(X_{1}=3)+49I(X_{1}=4)+\{\xi_{1}(1-A)+\xi_{2}A\}s_{j}+b_{0i}+b_{1i}s_{j}+e_{ij}$, where $b_{i}={\left(b_{0i},b_{1i}\right)}^{T}∼𝒩(0,D)$ independent of $e_{ij}∼𝒩(0,\sigma^{2})$. The outcome for subject i is then $Y_{i}=Z_{i5}$, the longitudinal measure at ${𝒯}_{F}$ weeks. As would be likely in practice, we included in X only the single baseline covariate $Z_{i1}$, the value of the longitudinal measure at time 0 and did not also include $X_{1}$, and we took the single time‐dependent covariate L(u) at time u to be the most recently observed value of the longitudinal measurements $Z_{ij}$. Under the null hypothesis, $\xi ={\left(-0.3,-0.3\right)}^{T}$; under the alternative, $\xi ={\left(-0.3,-0.18\right)}^{T}$ corresponding to $\beta =\beta_{0}=\beta_{A}=6.24$, for which $n_{\max}=300$ yields roughly 90% power at the final analysis. Results shown in the Supplemental Material demonstrate that the independent increments property holds approximately for all estimators. Here, the estimators ${\hat{\beta }}_{{𝒯}_{F}}(t)$ and ${\hat{\beta }}_{IPW}(t)$ are identical because $T={𝒯}_{F}$ for all subjects, so that both are based only on subjects followed for at least ${𝒯}_{F}$ weeks. SEs $SE\{{\hat{\beta }}_{{𝒯}_{F}}(t)\}$ are obtained from the routine formula for a difference in sample means assuming common treatment‐specific variance, while $SE\{{\hat{\beta }}_{IPW}(t)\}$ follows from the IPWCC influence function; these SEs are asymptotically equivalent but differ slightly for finite samples. Incorporation of the baseline covariate yields 10%‐20% gains in efficiency; further incorporation of the last outcome carried forward as a time‐dependent covariate leads to efficiency gains for ${\hat{\beta }}_{AIPW2}(t)$ of 34% to 47%.

Interim monitoring results are shown in Table 4 and are analogous to those in Tables 2 and 3. Under the null hypothesis, the Monte Carlo rejection probability for ${\hat{\beta }}_{AIPW2}(t)$ with Pocock boundaries exceeds slightly the nominal 0.025 level. Again, under the alternative, the AIPWCC estimators result in earlier expected sample sizes and stopping times.

**TABLE 4:** For Scenario 3 with continuous outcome, interim analysis performance using each estimator with O'Brien‐Fleming and Pocock stopping boundaries under (a) the null hypothesis β=0 and (b) the alternative β=6.24, with maximum sample size $n_{\max}=300$ and $t_{end}=208$ days

	P(reject)	MC E(SS)	MC E(Stop)	P(reject)	MC E(SS)	MC E(Stop)
	(a) Null hypothesis					
	O'Brien‐Fleming			Pocock		
${\hat{\beta }}_{{𝒯}_{F}}(t)$	0.026	299.9 (3.1)	207.4 (5.5)	0.025	298.6 (11.3)	206.2 (12.7)
${\hat{\beta }}_{IPW}(t)$	0.026	299.8 (3.4)	207.4 (5.8)	0.025	298.6 (11.5)	206.1 (12.9)
${\hat{\beta }}_{AIPW1}(t)$	0.025	299.9 (2.6)	207.5 (5.0)	0.026	298.6 (11.2)	206.2 (12.7)
${\hat{\beta }}_{AIPW2}(t)$	0.026	299.8 (4.0)	207.0 (7.4)	0.029	298.1 (13.2)	205.7 (14.5)

	(b) Alternative hypothesis					
	O'Brien‐Fleming			Pocock		
${\hat{\beta }}_{{𝒯}_{F}}(t)$	0.875	286.3 (26.0)	167.8 (30.6)	0.823	259.9 (45.2)	151.9 (41.9)
${\hat{\beta }}_{IPW}(t)$	0.876	286.0 (26.4)	167.4 (30.7)	0.826	259.1 (45.4)	151.1 (41.9)
${\hat{\beta }}_{AIPW1}(t)$	0.930	286.5 (25.5)	165.5 (28.9)	0.896	255.9 (45.2)	146.0 (39.6)
${\hat{\beta }}_{AIPW2}(t)$	0.930	265.9 (37.3)	146.7 (31.2)	0.892	239.8 (45.3)	133.5 (37.8)

*Note*: Entries are as in Table 2. The standard error for entries for P(reject) in (a) is ≈0.0016.

We remark that all scenarios reflect the general result that basing interim analyses on the proposed AIPWCC estimators leads to not only more efficient inferences but also, because of the increased precision, to a greater proportion of the total statistical information being available at each interim analysis time than would be available using the usual methods. This feature implies that O'Brien‐Fleming boundaries will be less conservative for the proposed estimators, leading to potential gains in expected sample size and stopping times.

## 6 APPLICATION

To demonstrate how use of the methods would proceed in practice as a trial progresses, we consider the setting of TESICO with ordinal categorical outcome, where the treatment effect of interest is the log odds ratio β in an assumed proportional odds model as in (2). Because this trial is ongoing, we cannot base this demonstration on data from the trial; accordingly, we present use of the methods for a simulated data set generated according to the first simulation scenario in Section 5 with β=log(1.5), which is based on this study. As in Section 5, the planned maximum sample size is $n_{\max}=602$, with full enrollment reached by $E_{\max}=240$ day. Interim analyses are planned at 150, 195, 240, and 285 days, with the final analysis to be conducted at $t_{end}=330$ days, at which time all $n_{\max}$ participants will have completed the trial with their outcomes ascertained. For definiteness, we use O'Brien‐Fleming stopping boundaries and focus on the null and alternative hypotheses $H_{0}:\beta_{0}=0$ vs $H_{A}:\beta_{0}>0$, with overall level of significance α=0.025.

Table 5 shows how the trial would proceed if the analyses were conducted at each interim analysis time t using each of the estimators ${\hat{\beta }}_{{𝒯}_{F}}(t)$, ${\hat{\beta }}_{IPW}(t)$, ${\hat{\beta }}_{AIPW1}(t)$, and ${\hat{\beta }}_{AIPW2}(t)$. For each estimator, the proportion of information at each of the interim analysis times was calculated as described in Section 4.2 and was used to obtain the stopping boundary. At the first interim analysis at 150 days, the proportion of information for the ML estimator ${\hat{\beta }}_{{𝒯}_{F}}(t)$, which uses only those subjects among the n(t) enrolled who have been followed for at least the maximum follow‐up time ${𝒯}_{F}$, is 0.257, whereas that for ${\hat{\beta }}_{AIPW2}(t)$ is 0.462, almost twice as large. This striking difference is reflected in the corresponding stopping boundaries: at the first interim analysis at 150 days, the test statistic based on ${\hat{\beta }}_{{𝒯}_{F}}(t)$ is 2.496, far from the boundary of 4.265, whereas that based on ${\hat{\beta }}_{AIPW2}(t)$ is 2.966, almost reaching the boundary of 3.099. Basing the analyses on ${\hat{\beta }}_{AIPW2}(t)$ results in sufficient evidence to stop the trial at the second interim analysis at 195 days with 487 subjects enrolled, while sufficient evidence to stop using ${\hat{\beta }}_{{𝒯}_{F}}(t)$ does not emerge until the fourth interim analysis at 285 days, with all $n_{\max}=602$ subjects enrolled. Basing the analyses on ${\hat{\beta }}_{IPW}(t)$ and ${\hat{\beta }}_{AIPW1}(t)$ results in stopping the trial at 240 days, with again all 602 planned subjects enrolled.

**TABLE 5:** Interim analysis results for analyses at time $\left(t_{1},\ldots ,t_{5})=(150,195,240,285,330\right)$ for the simulated TESICO trial; $n(t_{j})$ is the number of subjects enrolled at $t_{j}$

		${\hat{\beta }}_{{𝒯}_{F}}(t)$				${\hat{\beta }}_{IPW}(t)$			
$t_{j}$	$n(t_{j})$	Est (SE)	T	$p(t_{j})$	$b_{j}$	Est (SE)	T	p(t)	$b_{j}$
150	368	0.730 (0.292)	2.496	0.257	4.265	0.547 (0.230)	2.380	0.408	3.318
195	487	0.619 (0.224)	2.765	0.432	3.218	0.476 (0.193)	2.473	0.581	2.733
240	602	0.457 (0.187)	2.445	0.611	2.657	**0.423 (0.166)**	**2.551**	**0.785**	**2.313**
285	602	**0.459 (0.162)**	**2.828**	**0.809**	**2.277**	‐	‐	‐	‐
330	602	‐	‐	‐	‐	‐	‐	‐	‐

		${\hat{\beta }}_{AIPW1}(t)$				${\hat{\beta }}_{AIPW2}(t)$			
$t_{j}$	$n(t_{j})$	Est (SE)	T	p(t)	$b_{j}$	Est (SE)	T	p(t)	$b_{j}$
150	368	0.565 (0.218)	2.586	0.382	3.444	0.590 (0.199)	2.966	0.462	3.099
195	487	0.497 (0.182)	2.739	0.564	2.777	**0.532 (0.167)**	**3.185**	**0.670**	**2.521**
240	602	**0.409 (0.156)**	**2.615**	**0.757**	**2.362**	‐	‐	‐	‐
285	602	‐	‐	‐	‐	‐	‐	‐	‐
330	602	‐	‐	‐	‐	‐	‐	‐	‐

*Note*: For each of the estimators ${\hat{\beta }}_{{𝒯}_{F}}(t)$ (the ML estimator based on data from all subjects followed for at least the maximum follow‐up period ${𝒯}_{F}$ at t), the IPWCC estimator ${\hat{\beta }}_{IPW}(t)$, and the AIPWCC estimators ${\hat{\beta }}_{AIPW1}(t)$ and ${\hat{\beta }}_{AIPW2}(t)$, Est (SE) are the estimate $\hat{\beta }(t_{j})$ (standard error $SE\{\hat{\beta }(t_{j})\}$) at $t_{j}$, T is associated the Wald test statistic, $p(t_{j})$ is the proportion of information at $t_{j}$, and $b_{j}$ is the O'Brien‐Fleming stopping boundary. Entries are boldfaced at the interim analysis at which the trial would be stopped using the indicated estimator.

## 7 DISCUSSION

We have proposed a general framework for design and conduct of group sequential trials in the common situation where the outcome is known with certainty only after some time lag. The methods account for censoring at the time of an interim analysis and incorporate baseline and time‐dependent evolving covariate information to improve efficiency over standard analyses, facilitating earlier stopping with potentially smaller numbers of enrolled subjects. We have demonstrated analytically and empirically that the proposed test statistics possess the independent increments structure, so that standard methods and software for specifying stopping boundaries can be used. The methods can be applied under both information‐based monitoring and fixed‐sample monitoring strategies. For the latter, we have proposed the idea of effective sample size to characterize the proportion of information available at an interim analysis. Simulation studies demonstrate that the methods preserve the operating characteristics of a monitored trial and that substantial reductions in expected sample size and stopping time can be achieved.

As noted above, the proposed methodology is relevant in the large class of problems where the outcome would be known with certainty for all subjects at the final analysis. For some trials with possibly censored time‐to‐event outcome, interest may focus on the hazard ratio under the assumption of proportional hazards. Here, there is no prespecified, maximum follow‐up time ${𝒯}_{F}$ at which the outcome is known with certainty, so that the proposed framework is not applicable.

We have defined generically T as the lag time in ascertaining the outcome Y. In practice, there may be an administrative delay between the time an event related to the outcome has occurred and when that information is available in the trial database; for example, in TESICO, if a subject dies, there may be a delay before the time of death appears in the database. Our definition of lag time T should be taken to include such administrative delays.

The methods as presented are based on the assumption (12) that entry time is independent of all other variables, including baseline covariates X, which implies that at any interim analysis time t, $C(t)╨\{X,A,T,Y,\bar{L}(T)\}$. This assumption is made tacitly in any clinical trial that focuses on inference on an unconditional treatment effect parameter. If the distribution of X changes over the course of a trial, then (12) is violated, and, intuitively, subjects enrolled at the time of an interim analysis may not be representative of the population of interest at the final analysis. This is a general phenomenon and not unique to our methodology. If (12) is violated in this way, then the treatment effect parameter may not be static over time. Under these circumstances, conditional (on X) inference may be more appropriate; for example, as in the case of the conditional proportional odds model for ordinal categorical outcome in Section 2.1. Under the modified assumption $E╨\{A,T,Y,\bar{L}(T)\}|X$ (independence conditional on X), the proposed methods can be extended to support such conditional inference through incorporation of relevant influence functions and modeling of the censoring distribution as a function of X. In the Supplemental Material, we report on a simulation study based on Scenario 1 of Section 5 in which entry times are taken to be associated with X that suggests that the methods may enjoy some robustness to violations of (12).

## Supporting information


**Data S1**: Supporting Information

## Data Availability

Data sharing is not applicable to this article, as no data sets are generated or analyzed. The methods developed are proposed to enable future analyses of data from clinical trials, including ongoing trials from which the data are proprietary and not available. An R package, tLagInterim, implementing the methodology is available at the Comprehensive R Archive Network (CRAN) at https://cran.r‐project.org/package=tLagInterim.
