# Supplementary material for: Group sequential methods for interim monitoring of randomized clinical trials with time‐lagged outcome
Source: Stat Med. 2022 Sep 18;41(28):5517–36. doi: 10.1002/sim.9580 (PMC9825950; doi:10.1002/sim.9580)
Supplement: Supplementary file 1 — Data S1: Supporting Information [file SIM-41-5517-s001.pdf]

Received: Added at production | Revised: Added at production | Accepted: Added at production
DOI: xxx/xxxx

Supplemental Material for "Group sequential methods for interim monitoring of randomized clinical trials with time-lagged outcome"

Anastasios A. Tsiatis | Marie Davidian*

[1]Department of Statistics, North Carolina State University, North Carolina, USA

A | DEMONSTRATION OF INDEPENDENT INCREMENTS PROPERTY

For definiteness, we consider AIPWCC estimators $\widehat{\beta}(t)$ with influence function (13) with $f^{opt}(X)$ and $h^{opt}\{u, X, A, \bar{L}(u)\}$ in (19) substituted for $f(X)$ and $h\{u, X, A, \bar{L}(u)\}$, i.e., the efficient influence function; results for the IPWCC estimator follow from the argument.

We wish to describe the joint distribution of such estimators at interim analysis times $t_1 < \cdots < t_K$ (calendar time). It suffices to consider the bivariate distribution of $\{\widehat{\beta}(s), \widehat{\beta}(t)\}$, $s < t$. The large-sample properties of $\{\widehat{\beta}(s), \widehat{\beta}(t)\}$ are determined by the covariance matrix of the corresponding influence functions for $\widehat{\beta}(s)$ and $\widehat{\beta}(t)$.

Using

$$\frac{I(E \leq t)}{\text{pr}(E \leq t)} \frac{\Delta(t) m(Y, A, X; \alpha_0, \beta_0)}{\mathcal{K}_t\{U(t)\}} = \frac{I(E \leq t)}{\text{pr}(E \leq t)} \left[m(Y, A, X; \alpha_0, \beta_0) - \int_0^t \frac{dM_c^{(t)}(u)}{\mathcal{K}_t(u)} \{m(Y, A, X; \alpha_0, \beta_0) - \mu(m, u; \alpha_0, \beta_0)\} \right], \tag{A1}$$

$$\mu(h^{opt}, u) = E\left[h^{opt}\{u, X, A, \bar{L}(u)\} | T \geq u\right] = \frac{E\left[E\{m(Y, A, X; \alpha_0, \beta_0) | T \geq u, X, A, \bar{L}(u)\} | T \geq u\right]}{\mathcal{K}_t(u)}$$
$$= \frac{E\{m(Y, A, X; \alpha_0, \beta_0) | T \geq u\}}{\mathcal{K}_t(u)} = \frac{\mu(m, u; \alpha_0, \beta_0)}{\mathcal{K}_t(u)},$$

and denoting the available data at interim analysis times s and t by $\mathcal{O}^{(s)}$ and $\mathcal{O}^{(t)}$ as in (11), the efficient influence function for $\widehat{\beta}(t)$ is given by

$$m_t(\mathcal{O}^{(t)}; \alpha_0, \beta_0) = \frac{I(E \leq t)}{\text{pr}(E \leq t)} \Bigg(\{m(Y, A, X; \alpha_0, \beta_0) - (A - \pi) f^{opt}(X)\} \tag{A2}$$

$$- \int_0^t \frac{dM_c^{(t)}(u)}{\mathcal{K}_t(u)} \left[m(Y, A, X; \alpha_0, \beta_0) - E\{m(Y, A, X; \alpha_0, \beta_0) | T \geq u, X, A, \bar{L}(u)\}\right] \Bigg). \tag{A3}$$

Similarly, denote the efficient influence function for $\widehat{\beta}(s)$ as $m_s(\mathcal{O}^{(s)}; \alpha_0, \beta_0)$. For brevity, we suppress dependence of these influence functions on α_0, β_0 henceforth.

To demonstrate the independent increments property, it suffices to show that

$$E\{m_s(\mathcal{O}^{(s)}) m_t(\mathcal{O}^{(t)})\} = \text{var}\{m_t(\mathcal{O}^{(t)})\} = E\{m_t(\mathcal{O}^{(t)})^2\}. \tag{A4}$$

To this end, recalling that $C(t) = t - E$, it is convenient to define

$$\widetilde{N}_c^{(t)}(u) = I\{C(t) \leq u\} = I(E \geq t - u), \quad \widetilde{\mathcal{Y}}^{(t)}(u) = I\{C(t) \geq u\} = I(E \leq t - u),$$

and note that $\mathcal{K}_t(u) = \text{pr}\{C(t) \geq u | E \leq t\} = \text{pr}(E \leq t-u | E \leq t)$. Denoting the distribution of E by $Q(u) = \text{pr}(E \leq u)$, with density $q(u)$, and recalling that $\Lambda_c^{(t)}(u) = -\log\{\mathcal{K}_t(u)\}$, it follows that

$$\mathcal{K}_t(u) = Q(t-u)/Q(t), \quad d\Lambda_c^{(t)}(u) = \{q(t-u)/Q(t-u)\}\, du.$$

Thus, if we write

$$d\widetilde{M}(t-u) = d\widetilde{N}_c^{(t)}(u) - d\Lambda_c^{(t)}(u)\widetilde{\mathcal{Y}}^{(t)}(u) = -dI(E \geq t-u) - \frac{q(t-u)}{Q(t-u)} I(E \leq t-u)\, du, \tag{A5}$$

then using $N_c^{(t)}(u) = I\{C(t) \leq u, T \geq u\}$ and $\mathcal{Y}^{(t)}(u) = I\{C(t) \geq u, T \geq u\}$, the martingale integral (A3) can be written as

$$-\int_0^t \frac{d\widetilde{M}(t-u) I(T \geq u)}{Q(t-u)/Q(t)} \big[m(Y,A,X) - E\{m(Y,A,X) | T \geq u, X, A, \bar{L}(u)\}\big]$$

$$= -\int_0^t \frac{d\widetilde{M}(x) I(T \geq t-x)}{Q(x)/Q(t)} \big[m(Y,A,X) - E\{m(Y,A,X) | T \geq t-x, X, A, \bar{L}(t-x)\}\big] \tag{A6}$$

with change of variable $x = t-u$. If we define the filtration $\mathcal{F}(x)$ to be the sigma algebra generated by $\{I(E \geq x), EI(E \geq x), Y, A, X, \bar{L}(T)\}$, then the integral (A6) is the realization of a $\mathcal{F}(x)$-measurable martingale process. Note that the filtration $\mathcal{F}(x)$ defines information about E to the right (after) x rather than as for the usual filtration that defines information to the left (before) x. Thus, (A3) can be written as

$$-\frac{I(E \leq t)}{Q(t)} \int_0^t \frac{d\widetilde{M}(x) I(T \geq t-x)}{Q(x)/Q(t)} \big[m(Y,A,X) - E\{m(Y,A,X) | T \geq t-x, X, A, \bar{L}(t-x)\}\big]$$

$$= -\int_0^t \frac{d\widetilde{M}(x) I(T \geq t-x)}{Q(x)} \big[\{m(Y,A,X) - E\{m(Y,A,X) | T \geq t-x, X, A, \bar{L}(t-x)\}\big],$$

and the variance of (A3) is

$$E\left(\int_0^t \frac{q(x)\,dx/Q(x)}{Q^2(x)} I(E \leq x) I(T \geq t-x) \big[m(Y,A,X) - E\{m(Y,A,X) | T \geq t-x, X, A, \bar{L}(t-x)\}\big]^2\right)$$

$$= \int_0^t \frac{q(x)\,dx}{Q^2(x)} E\left(\big[m(Y,A,X) - E\{m(Y,A,X) | T \geq t-x, X, A, \bar{L}(t-x)\}\big]^2 I(T \geq t-x)\right).$$

Consequently,

$$\begin{aligned} \text{var}\{m_t(\mathcal{O}^{(t)})\} = {} & \frac{\text{var}\{m(Y,A,X) - (A-\pi) f^{opt}(X)\}}{Q(t)} \\ & + \int_0^t \frac{q(x)\,dx}{Q^2(x)} E\left(\big[m(Y,A,X) - E\{m(Y,A,X) | T \geq t-x, X, A, \bar{L}(t-x)\}\big]^2 I(T \geq t-x)\right), \end{aligned} \tag{A7}$$

and similarly for $\text{var}\{m_s(\mathcal{O}^{(s)})\}$.

From (A4), we thus wish to show that $E\{m_s(\mathcal{O}^{(s)}) m_t(\mathcal{O}^{(t)})\}$ is equal to $\text{var}\{m_t(\mathcal{O}^{(t)})\}$ in (A7). Using the preceding developments, we can write

$$m_t(\mathcal{O}^{(t)}) = \frac{I(E \leq t)}{Q(t)} \{m(Y,A,X) - (A-\pi) f^{opt}(X)\} \tag{A8}$$

$$-\int_0^t \frac{d\widetilde{M}(x)}{Q(x)} \big[\{m(Y,A,X) - E\{m(Y,A,X) | T \geq t-x, X, A, \bar{L}(t-x)\}\big] I(T \geq t-x) \tag{A9}$$

$$m_s(\mathcal{O}^{(s)}) = \frac{I(E \le s)}{Q(s)}\{m(Y,A,X) - (A-\pi)f^{opt}(X)\} \tag{A10}$$

$$- \int_0^s \frac{d\widetilde{M}(x)}{Q(x)}\big[\{m(Y,A,X) - E\{m(Y,A,X)|T \ge s-x, X, A, \bar{L}(s-x)\}\big] I(T \ge s-x); \tag{A11}$$

accordingly,

$$E\{m_s(\mathcal{O}^{(s)})m_t(\mathcal{O}^{(t)})\} = (A8)\times(A10) + (A8)\times(A11) + (A9)\times(A10) + (A9)\times(A11).$$

We consider each of these terms in turn.

Using $s \le t$, it is straightforward that

$$E\{(A8)\times(A10)\} = E\left[\frac{I(E \le s)}{Q(s)Q(t)}\{m(Y,A,X) - (A-\pi)f^{opt}(X)\}^2\right]$$
$$= \frac{\text{var}\{m(Y,A,X) - (A-\pi)f^{opt}(X)\}}{Q(t)}. \tag{A12}$$

Similarly,

$$E\{(A8)\times(A11)\} = E\Bigg(-\frac{I(E \le s)}{Q(t)}\{m(Y,A,X) - (A-\pi)f^{opt}(X)\}$$
$$\times \int_0^s \frac{d\widetilde{M}(x)}{Q(x)}\big[m(Y,A,X) - E\{m(Y,A,X)|T \ge s-x, X, A, \bar{L}(s-x)\}\big] I(T \ge s-x)\Bigg)$$
$$= E\Bigg(\frac{\{m(Y,A,X) - (A-\pi)f^{opt}(X)\}}{Q(t)}$$
$$\times \int_0^s \frac{d\widetilde{M}(x)}{Q(x)}\big[m(Y,A,X) - E\{m(Y,A,X)|T \ge s-x, X, A, \bar{L}(s-x)\}\big] I(T \ge s-x)\Bigg)$$
$$= 0 \tag{A13}$$

because $\{m(Y,A,X) - (A-\pi)f^{opt}(X)\}$ is $\mathcal{F}(s)$-predictable, so that the expectation is zero by the martingale property of stochastic integrals.

By the martingale property,

$$E\{(A9)\times(A11)\} = \int_0^s \frac{q(x)dx}{Q^2(x)} E\Big(\big[m(Y,A,X) - E\{m(Y,A,X)|T \ge t-x, X, A, \bar{L}(t-x)\}\big]$$
$$\times \big[m(Y,A,X) - E\{m(Y,A,X)|T \ge s-x, X, A, \bar{L}(s-x)\}\big] I(T \ge t-x)\Big). \tag{A14}$$

The expectation in the intetgral in (A14) can be written as

$$E\left(\big[m(Y,A,X) - E\{m(Y,A,X)|T \ge t-x, X, A, \bar{L}(t-x)\}\big]^2 I(T \ge t-x)\right)$$
$$- E\Big(\big[E\{m(Y,A,X)|T \ge t-x, X, A, \bar{L}(t-x)\} - E\{m(Y,A,X)|T \ge s-x, X, A, \bar{L}(s-x)\}\big] \tag{A15}$$
$$\times \big[m(Y,A,X) - E\{m(Y,A,X)|T \ge t-x, X, A, \bar{L}(t-x)\}\big] I(T \ge t-x)\Big).$$

The difference of conditional expectations in brackets in (A15) is some function $g\{X, A, \bar{L}(t-x)\}$, in which case the last two lines of (A15) can be written as

$$E\Big(g\{X,A,\bar{L}(t-x)\}\big[m(Y,A,X) - E\{m(Y,A,X)|T \ge t-x, X, A, \bar{L}(t-x)\}\big] I(T \ge t-x)\Big)$$
$$= E\left\{E\left(g\{X,A,\bar{L}(t-x)\}\big[m(Y,A,X) - E\{m(Y,A,X)|T \ge t-x, X, A, \bar{L}(t-x)\}\big] I(T \ge t-x)\right) \mid T \ge t-x, X, A, \bar{L}(t-x)\right\}$$
$$= E\left(g\{X,A,\bar{L}(t-x)\}\big[E\{m(Y,A,X)|T \ge t-x, X, A, \bar{L}(t-x)\} - E\{m(Y,A,X)|T \ge t-x, X, A, \bar{L}(t-x)\}\big] I(T \ge t-x)\right)$$
$$= 0.$$

It follows that

$$E\{(A9)\times(A11)\} = \int_0^s \frac{q(x)dx}{Q^2(x)} E\left(\left[m(Y,A,X) - E\{m(Y,A,X)|T \geq t-x, X, A, \bar{L}(t-x)\}\right]^2 I(T \geq t-x)\right). \qquad \text{(A16)}$$

Finally, consider

$$\begin{aligned} E\{(A9)\times(A10)\} = -E\Bigg(& \frac{I(E \leq s)}{Q(s)}\{m(Y,A,X) - (A-\pi)f^{opt}(X)\} \\ & \times \int_0^t \frac{d\widetilde{M}(x)}{Q(x)}\left[\{m(Y,A,X) - E\{m(Y,A,X)|T \geq t-x, X, A, \bar{L}(t-x)\}\right] I(T \geq t-x)\Bigg). \end{aligned} \qquad \text{(A17)}$$

We can write (A17) as

$$\begin{aligned} -E\Bigg(& \frac{I(E \leq s)}{Q(s)}\{m(Y,A,X) - (A-\pi)f^{opt}(X)\} \\ & \times \int_0^s \frac{d\widetilde{M}(x)}{Q(x)}\left[\{m(Y,A,X) - E\{m(Y,A,X)|T \geq t-x, X, A, \bar{L}(t-x)\}\right] I(T \geq t-x)\Bigg) \end{aligned} \qquad \text{(A18)}$$

$$\begin{aligned} -E\Bigg(& \frac{I(E \leq s)}{Q(s)}\{m(Y,A,X) - (A-\pi)f^{opt}(X)\} \\ & \times \int_s^t \frac{d\widetilde{M}(x)}{Q(x)}\left[\{m(Y,A,X) - E\{m(Y,A,X)|T \geq t-x, X, A, \bar{L}(t-x)\}\right] I(T \geq t-x)\Bigg). \end{aligned} \qquad \text{(A19)}$$

Because $\{m(Y,A,X) - (A-\pi)f^{opt}(X)\}$ is $\mathcal{F}(s)$-predictable, (A18) is equal to zero. Thus, consider (A19). Recalling from (A5) that

$$d\widetilde{M}(x) = -dI(E \geq x) - \frac{q(x)}{Q(x)} I(E \leq x)dx$$

and noting that for $x \geq s$

$$I(E \leq s)\{-dI(E \geq x)\} = 0, \quad I(E \leq s)I(E \leq x) = I(E \leq s),$$

it follows that (A19) can be written as

$$\begin{aligned} & E\left(\int_s^t \frac{\{m(Y,A,X) - (A-\pi)f^{opt}(X)\}}{Q(s)} \frac{q(x)dx}{Q^2(x)} I(E \leq s)\left[\{m(Y,A,X) - E\{m(Y,A,X)|T \geq t-x, X, A, \bar{L}(t-x)\}\right] I(T \geq t-x)\right) \\ & = \int_s^t \frac{q(x)dx}{Q^2(x)} E\left(\{m(Y,A,X) - (A-\pi)f^{opt}(X)\}\left[\{m(Y,A,X) - E\{m(Y,A,X)|T \geq t-x, X, A, \bar{L}(t-x)\}\right] I(T \geq t-x)\right). \end{aligned}$$

(A20)

Write the expectation in the integrand of (A20) as

$$\begin{aligned} & E\left(\left[\{m(Y,A,X) - E\{m(Y,A,X)|T \geq t-x, X, A, \bar{L}(t-x)\}\right]^2 I(T \geq t-x)\right) \\ & + E\left(\left[E\{m(Y,A,X)|T \geq t-x, X, A, \bar{L}(t-x)\} - (A-\pi)f^{opt}(X)\right]\right. \\ & \left.\times \left[\{m(Y,A,X) - E\{m(Y,A,X)|T \geq t-x, X, A, \bar{L}(t-x)\}\right] I(T \geq t-x)\right). \end{aligned} \qquad \text{(A21)}$$

Because the term in brackets in (A21) is some function $g^*\{X, A, \bar{L}(t-x)\}$, say, it follows by an argument similar to that above that the last two lines are equal to zero, and thus (A17) is equal to

$$E\{(A9)\times(A10)\} = \int_s^t \frac{q(x)dx}{Q^2(x)} E\left(\left[\{m(Y,A,X) - E\{m(Y,A,X)|T \geq t-x, X, A, \bar{L}(t-x)\}\right]^2 I(T \geq t-x)\right). \qquad \text{(A22)}$$

Combining the results in (A12), (A13), (A16), and (A22) demonstrates the desired result that

$$E\{m_s(\mathcal{O}^{(s)})m_t(\mathcal{O}^{(t)})\} = \frac{\text{var}\{m(Y,A,X) - (A-\pi)f^{opt}(X)\}}{Q(t)} + \int_0^t \frac{q(x)dx}{Q^2(x)} E\left(\left[m(Y,A,X) - E\{m(Y,A,X)|T \geq t-x, X, A, \bar{L}(t-x)\}\right]^2 I(T \geq t-x)\right),$$

which is (A7).

Note that the influence function for the IPWCC estimator solving (15) is given by (13) with $f(X) = h\{u, X, A, \bar{L}(u)\} \equiv 0$. Using the equality (A1) and the definition of $\widetilde{M}(x)$, the influence function for the IPWCC estimator can be written as

$$m_t^{IPW}(\mathcal{O}^{(t)}) = \frac{I(E \leq t)}{Q(t)} m(Y,A,X) - \int_0^t \frac{d\widetilde{M}(x)}{Q(x)} \left[m(Y,A,X) - E\{m(Y,A,X)|T \geq t-x\}\right] I(T \geq t-x).$$

That $E\{m_s^{IPW}(\mathcal{O}^{(s)})m_t^{IPW}(\mathcal{O}^{(s)})\} = \text{var}\{m_t^{IPW}(\mathcal{O}^{(t)})\}$ follows by an argument analogous to that above, demonstrating that the IPWCC estimator also has the independent increments property.

In the practical implementation discussed in Section 4, the optimal choices $f^{opt}(X)$ and $h^{opt}\{u, X, A, \bar{L}(u)\}$ are approximated using linear combinations of basis functions. Accordingly, the resulting AIPWCC estimators obtained via the two-step algorithm may not be fully efficient and thus are not guaranteed to have the independent increments property. However, as demonstrated in our simulation studies, because the approximations to $f^{opt}(X)$ and $h^{opt}\{u, X, A, \bar{L}(u)\}$ are often quite good, the estimators themselves are good approximations to the efficient estimator and thus exhibit behavior very close to that of independent increments, so that the operating characteristics of the trial are preserved.

B | ADDITIONAL SIMULATION RESULTS

Simulation Scenario 1: Ordinal Categorical Outcome: Under the null hypothesis, based on 10000 Monte Carlo data sets, the Monte Carlo sample covariance matrices of $\{\hat{\beta}(t_1), \dots, \hat{\beta}(t_4), \hat{\beta}(t_{end})\}$, where $\hat{\beta}(t)$ is each of $\hat{\beta}_{\mathcal{T}_F}(t)$, $\hat{\beta}_{IPW}(t)$, $\hat{\beta}_{AIPW1}(t)$, and $\hat{\beta}_{AIPW2}(t)$, are given by

```
betahat_Tf
      [,1]  [,2]  [,3]  [,4]  [,5]
[1,] 0.086 0.049 0.034 0.026 0.021
[2,] 0.049 0.049 0.034 0.026 0.021
[3,] 0.034 0.034 0.034 0.026 0.021
[4,] 0.026 0.026 0.026 0.026 0.021
[5,] 0.021 0.021 0.021 0.021 0.021

betahat_IPW
[1,] 0.054 0.036 0.027 0.023 0.022
[2,] 0.036 0.036 0.027 0.023 0.022
[3,] 0.027 0.027 0.028 0.023 0.022
[4,] 0.023 0.023 0.023 0.023 0.022
[5,] 0.022 0.022 0.022 0.022 0.022

betahat_AIPW1
      [,1]  [,2]  [,3]  [,4]  [,5]
[1,] 0.049 0.031 0.023 0.019 0.018
[2,] 0.031 0.032 0.023 0.019 0.018
[3,] 0.023 0.023 0.024 0.020 0.019
[4,] 0.019 0.019 0.020 0.020 0.018
[5,] 0.018 0.018 0.019 0.018 0.018

betahat_AIPW2
      [,1]  [,2]  [,3]  [,4]  [,5]
[1,] 0.041 0.027 0.022 0.019 0.019
[2,] 0.027 0.028 0.021 0.019 0.018
[3,] 0.022 0.021 0.022 0.019 0.019
[4,] 0.019 0.019 0.019 0.019 0.018
[5,] 0.019 0.018 0.019 0.018 0.018
```

Under the alternative $\beta_A = \log(1.5)$, the analogous Monte Carlo sample covariance matrices are

```
betahat_Tf
      [,1]  [,2]  [,3]  [,4]  [,5]
[1,] 0.087 0.048 0.034 0.026 0.021
[2,] 0.048 0.049 0.034 0.026 0.021
[3,] 0.034 0.034 0.034 0.027 0.022
[4,] 0.026 0.026 0.027 0.027 0.022
[5,] 0.021 0.021 0.022 0.022 0.022
```

```
betahat_IPW
      [,1]  [,2]  [,3]  [,4]  [,5]
[1,] 0.055 0.036 0.028 0.023 0.022
[2,] 0.036 0.036 0.028 0.023 0.022
[3,] 0.028 0.028 0.028 0.024 0.022
[4,] 0.023 0.023 0.024 0.024 0.022
[5,] 0.022 0.022 0.022 0.022 0.022

betahat_AIPW1
     [,1]  [,2]  [,3]  [,4]  [,5]
[1,] 0.050 0.032 0.024 0.020 0.019
[2,] 0.032 0.032 0.024 0.020 0.019
[3,] 0.024 0.024 0.025 0.020 0.019
[4,] 0.020 0.020 0.020 0.020 0.019
[5,] 0.019 0.019 0.019 0.019 0.019

betahat_AIPW2
[1,] 0.042 0.027 0.022 0.019 0.019
[2,] 0.027 0.029 0.022 0.019 0.019
[3,] 0.022 0.022 0.022 0.019 0.019
[4,] 0.019 0.019 0.019 0.019 0.019
[5,] 0.019 0.019 0.019 0.019 0.019
```

These results clearly demonstrate that the independent increments property holds approximately for all estimators.

Simulation Scenario 2: Binary Outcome: Under the null hypothesis, based on 10000 Monte Carlo data sets, the Monte Carlo sample covariance matrices of $\{\widehat{\beta}(t_1), \ldots, \widehat{\beta}(t_4), \widehat{\beta}(t_{end})\}$, where $\widehat{\beta}(t)$ is each of $\widehat{\beta}_{\mathcal{T}_F}(t)$, $\widehat{\beta}_{IPW}(t)$, $\widehat{\beta}_{AIPW1}(t)$, and $\widehat{\beta}_{AIPW2}(t)$, are given by

```
betahat_Tf
      [,1]  [,2]  [,3]  [,4]  [,5]
[1,] 0.039 0.022 0.015 0.012 0.009
[2,] 0.022 0.021 0.015 0.011 0.009
[3,] 0.015 0.015 0.015 0.011 0.009
[4,] 0.012 0.011 0.011 0.011 0.009
[5,] 0.009 0.009 0.009 0.009 0.009

betahat_IPW
      [,1]  [,2]  [,3]  [,4]  [,5]
[1,] 0.018 0.013 0.011 0.009 0.009
[2,] 0.013 0.013 0.010 0.009 0.009
[3,] 0.011 0.010 0.010 0.009 0.009
[4,] 0.009 0.009 0.009 0.009 0.009
[5,] 0.009 0.009 0.009 0.009 0.009

betahat_AIPW1
      [,1]  [,2]  [,3]  [,4]  [,5]
[1,] 0.017 0.012 0.009 0.008 0.008
[2,] 0.012 0.012 0.009 0.008 0.008
[3,] 0.009 0.009 0.009 0.008 0.008
[4,] 0.008 0.008 0.008 0.008 0.008
[5,] 0.008 0.008 0.008 0.008 0.008
```

```
betahat_AIPW2
      [,1]  [,2]  [,3]  [,4]  [,5]
[1,] 0.017 0.012 0.009 0.008 0.008
[2,] 0.012 0.012 0.009 0.008 0.008
[3,] 0.009 0.009 0.009 0.008 0.008
[4,] 0.008 0.008 0.008 0.008 0.008
[5,] 0.008 0.008 0.008 0.008 0.008
```

Under the alternative $\beta_A = \log(0.247/0.33) = -0.290$, the analogous Monte Carlo sample covariance matrices are

```
betahat_Tf
      [,1]  [,2]  [,3]  [,4]  [,5]
[1,] 0.049 0.027 0.019 0.015 0.012
[2,] 0.027 0.027 0.019 0.014 0.012
[3,] 0.019 0.019 0.019 0.014 0.011
[4,] 0.015 0.014 0.014 0.014 0.011
[5,] 0.012 0.012 0.011 0.011 0.011

betahat_IPW
      [,1]  [,2]  [,3]  [,4]  [,5]
[1,] 0.023 0.017 0.013 0.012 0.012
[2,] 0.017 0.017 0.013 0.012 0.011
[3,] 0.013 0.013 0.013 0.011 0.011
[4,] 0.012 0.012 0.011 0.011 0.011
[5,] 0.012 0.011 0.011 0.011 0.011

betahat_AIPW1
      [,1]  [,2]  [,3] [,4] [,5]
[1,] 0.022 0.015 0.012 0.010 0.010
[2,] 0.015 0.015 0.012 0.010 0.010
[3,] 0.012 0.012 0.012 0.010 0.010
[4,] 0.010 0.010 0.010 0.010 0.010
[5,] 0.010 0.010 0.010 0.010 0.010

betahat_AIPW2
      [,1]  [,2]  [,3] [,4] [,5]
[1,] 0.022 0.015 0.012 0.010 0.010
[2,] 0.015 0.015 0.012 0.010 0.010
[3,] 0.012 0.012 0.012 0.010 0.010
[4,] 0.010 0.010 0.010 0.010 0.010
[5,] 0.010 0.010 0.010 0.010 0.010
```

These results clearly demonstrate that the independent increments property holds approximately for all estimators.

Table B1 presents performance of the estimators under the null and alternative hypotheses.

TABLE B1 For Scenario 2 with binary outcome, performance of estimators for β under (a) the null hypothesis $\beta = 0$ and (b) the alternative $\beta = \log(0.247/0.33) = -0.290$ at each interim analysis time $(t_1, \dots, t_4) = (150, 195, 240, 285)$ days and at the final analysis at $t_{end} = 330$ days MC Mean is the mean of 10000 Monte Carlo estimates; MC SD is the Monte Carlo standard deviation, Ave MC SE is the mean of Monte Carlo standard errors, and MSE ratio is the ratio of Monte Carlo mean square error for $\widehat{\beta}_{\mathcal{T}_F}(t)$ divided by that for the indicated estimator.

	MC Mean	MC SD	Ave MC SE	MSE ratio	MC Mean	MC SD	Ave MC SE	MSE ratio
	**(a) Null Hypothesis**							
	$\widehat{\beta}_{\mathcal{T}_F}(t)$				$\widehat{\beta}_{IPW}(t)$			
t_1	0.003	0.197	0.193	1.000	0.000	0.136	0.134	2.097
t_2	0.000	0.146	0.145	1.000	0.000	0.115	0.114	1.600
t_3	0.000	0.122	0.121	1.000	-0.001	0.102	0.101	1.433
t_4	0.000	0.106	0.106	1.000	0.000	0096	0.095	1.237
t_{end}	0.000	0.096	0.095	1.000	0.000	0.096	0.095	1.000
	$\widehat{\beta}_{AIPW1}(t)$				$\widehat{\beta}_{AIPW2}(t)$			
t_1	0.000	0.130	0.128	2.302	0.001	0.130	0.128	2.300
t_2	0.000	0.110	0.109	1.761	0.000	0.110	0.109	1.759
t_3	-0.001	0.097	0.097	1.587	-0.001	0.097	0.096	1.591
t_4	0.000	0.090	0.090	1.389	0.000	0.090	0.090	1.389
t_{end}	0.000	0.090	0.090	1.123	0.000	0.090	0.090	1.123
	**(b) Alternative Hypothesis**							
	$\widehat{\beta}_{\mathcal{T}_F}(t)$				$\widehat{\beta}_{IPW}(t)$			
t_1	-0.291	0.220	0.216	1.000	-0.292	0.153	0.151	2.072
t_2	-0.291	0.164	0.162	1.000	-0.291	0.130	0.129	1.591
t_3	-0.292	0.136	0.135	1.000	-0.291	0.115	0.114	1.412
t_4	-0.291	0.119	0.118	1.000	-0.290	0.107	0.107	1.242
t_{end}	-0.290	0.107	0.107	1.000	-0.290	0.107	0.107	1.000
	$\widehat{\beta}_{AIPW1}(t)$				$\widehat{\beta}_{AIPW2}(t)$			
t_1	-0.291	0.147	0.146	2.246	-0.291	0.147	0.145	2.249
t_2	-0.291	0.124	0.123	1.734	-0.291	0.124	0.123	1.736
t_3	-0.291	0.110	0.109	1.545	-0.291	0.109	0.109	1.548
t_4	-0.290	0.102	0.102	1.377	-0.290	0.102	0.101	1.373
t_{end}	-0.290	0.101	0.101	1.109	-0.290	0.101	0.101	1.109

Simulation Scenario 3: Continuous Outcome: Under the null hypothesis, based on 10000 Monte Carlo data sets, the Monte Carlo sample covariance matrices of $\{\hat{\beta}(t_1), \ldots, \hat{\beta}(t_4), \hat{\beta}(t_{end})\}$, where $\hat{\beta}(t)$ is each of $\hat{\beta}_{T_F}(t)$, $\hat{\beta}_{IPW}(t)$, $\hat{\beta}_{AIPW1}(t)$, and $\hat{\beta}_{AIPW2}(t)$, are given by

```
betahat_Tf
      [,1] [,2] [,3] [,4] [,5]
[1,] 11.67 7.84 5.85 4.65 3.85
[2,]  7.84 7.89 5.87 4.69 3.90
[3,]  5.85 5.87 5.83 4.65 3.87
[4,]  4.65 4.69 4.65 4.63 3.86
[5,]  3.85 3.90 3.87 3.86 3.88

betahat_IPW
      [,1] [,2] [,3] [,4] [,5]
[1,] 11.67 7.84 5.85 4.65 3.85
[2,]  7.84 7.89 5.87 4.69 3.90
[3,]  5.85 5.87 5.83 4.65 3.87
[4,]  4.65 4.69 4.65 4.63 3.86
[5,]  3.85 3.90 3.87 3.86 3.88

betahat_AIPW1
      [,1] [,2] [,3] [,4] [,5]
[1,] 10.65 6.81 4.82 3.82 3.14
[2,]  6.81 7.08 5.04 3.86 3.19
[3,]  4.82 5.04 5.13 3.96 3.16
[4,]  3.82 3.86 3.96 3.94 3.16
[5,]  3.14 3.19 3.16 3.16 3.17

betahat_AIPW2
     [,1] [,2] [,3] [,4] [,5]
[1,] 7.92 5.47 4.27 3.50 3.17
[2,] 5.47 5.44 4.17 3.49 3.16
[3,] 4.27 4.17 4.15 3.48 3.16
[4,] 3.50 3.49 3.48 3.46 3.16
[5,] 3.17 3.16 3.16 3.16 3.17
```

Under the alternative $\beta_A = 6.24$, the analogous Monte Carlo sample covariance matrices are

```
betahat_Tf
      [,1] [,2] [,3] [,4] [,5]
[1,] 11.71 7.87 5.88 4.67 3.86
[2,]  7.87 7.92 5.90 4.71 3.91
[3,]  5.88 5.90 5.85 4.67 3.89
[4,]  4.67 4.71 4.67 4.65 3.88
[5,]  3.86 3.91 3.89 3.88 3.89
```

```
betahat_IPW
      [,1] [,2] [,3] [,4] [,5]
[1,] 11.71 7.87 5.88 4.67 3.86
[2,]  7.87 7.92 5.90 4.71 3.91
[3,]  5.88 5.90 5.85 4.67 3.89
[4,]  4.67 4.71 4.67 4.65 3.88
[5,]  3.86 3.91 3.89 3.88 3.89

betahat_AIPW1
      [,1] [,2] [,3] [,4] [,5]
[1,] 10.69 6.84 4.84 3.83 3.15
[2,]  6.84 7.10 5.06 3.87 3.20
[3,]  4.84 5.06 5.15 3.97 3.18
[4,]  3.83 3.87 3.97 3.95 3.17
[5,]  3.15 3.20 3.18 3.17 3.18

betahat_AIPW2
     [,1] [,2] [,3] [,4] [,5]
[1,] 7.94 5.49 4.29 3.52 3.18
[2,] 5.49 5.46 4.18 3.51 3.17
[3,] 4.29 4.18 4.16 3.49 3.18
[4,] 3.52 3.51 3.49 3.47 3.18
[5,] 3.18 3.17 3.18 3.18 3.18
```

Table B2 presents performance of the estimators under the null and alternative hypotheses.

TABLE B2 For Scenario 3 with continuous outcome, performance of estimators for β under (a) the null hypothesis $\beta = 0$ and (b) the alternative $\beta = 6.24$ at each interim analysis time $(t_1, \dots, t_4) = (104, 130, 156, 182)$ days and at the final analysis at $t_{end} = 208$ days MC Mean is the mean of 10000 Monte Carlo estimates; MC SD is the Monte Carlo standard deviation, Ave MC SE is the mean of Monte Carlo standard errors, and MSE ratio is the ratio of Monte Carlo mean square error for $\widehat{\beta}_{\mathcal{T}_F}(t)$ divided by that for the indicated estimator.

	MC Mean	MC SD	Ave MC SE	MSE ratio	MC Mean	MC SD	Ave MC SE	MSE ratio
**(a) Null Hypothesis**								
	$\widehat{\beta}_{\mathcal{T}_F}(t)$				$\widehat{\beta}_{IPW}(t)$			
t_1	0.012	3.416	3.415	1.000	0.012	3.416	3.380	1.000
t_2	0.010	2.809	2.781	1.000	0.010	2.809	2.763	1.000
t_3	-0.001	2.414	2.407	1.000	-0.001	2.414	2.395	1.000
t_4	-0.005	2.152	2.151	1.000	-0.005	2.152	2.142	1.000
t_{end}	0.005	1.969	1.962	1.000	0.005	1.969	1.956	1.000
	$\widehat{\beta}_{AIPW1}(t)$				$\widehat{\beta}_{AIPW2}(t)$			
t_1	0.007	3.264	3.222	1.095	-0.009	2.813	2.721	1.474
t_2	-0.006	2.660	2.609	1.115	-0.003	2.332	2.284	1.452
t_3	0.002	2.265	2.247	1.136	0.001	2.037	2.014	1.405
t_4	-0.001	1.985	1.975	1.176	-0.001	1.859	1.839	1.340
t_{end}	0.008	1.780	1.772	1.225	0.008	1.780	1.772	1.225
**(b) Alternative Hypothesis**								
	$\widehat{\beta}_{\mathcal{T}_F}(t)$				$\widehat{\beta}_{IPW}(t)$			
t_1	6.230	3.422	3.421	1.000	6.230	3.422	3.386	1.000
t_2	6.208	2.815	2.786	1.000	6.208	2.815	2.768	1.000
t_3	6.216	2.419	2.411	1.000	6.216	2.419	2.399	1.000
t_4	6.213	2.157	2.154	1.000	6.213	2.157	2.146	1.000
t_{end}	6.223	1.973	1.966	1.000	6.223	1.973	1.959	1.000
	$\widehat{\beta}_{AIPW1}(t)$				$\widehat{\beta}_{AIPW2}(t)$			
t_1	6.225	3.269	3.227	1.096	6.210	2.818	2.726	1.474
t_2	6.212	2.665	2.613	1.115	6.216	2.336	2.288	1.452
t_3	6.220	2.269	2.250	1.137	6.219	2.041	2.017	1.405
t_4	6.217	1.989	1.979	1.176	6.217	1.863	1.842	1.341
t_{end}	6.226	1.783	1.775	1.225	6.226	1.783	1.775	1.225

C SIMULATION UNDER ENROLLMENT DEPENDING ON X

We consider Scenario 1, with ordinal categorical outcome. Instead of taking the enrollment process to be uniform on the calendar time interval $[0, E_{max}]$, we generated entry time E according to a proportional hazards model to induce an association between E and X. Specifically, with all other variables generated as in Section 5 of the main paper, we generated E as

$$E = (1 - W^{\exp(\zeta X)})E_{max},$$

where $W \sim U(0,1)$ and $\zeta = -0.5$. Here, the baseline survival distribution for E is uniform on $[0, E_{max}]$. Taking $\zeta = -0.5$ with X as generated in Section 5 results in the means of X at $E = 0$ and $E = 240$ spanning the interquartile range of X.

Under the null hypothesis, based on 10000 Monte Carlo data sets, the Monte Carlo sample covariance matrices of $\{\hat{\beta}(t_1), \dots, \hat{\beta}(t_4), \hat{\beta}(t_{end})\}$, where $\hat{\beta}(t)$ is each of $\hat{\beta}_{\mathcal{T}_F}(t)$, $\hat{\beta}_{IPW}(t)$, $\hat{\beta}_{AIPW1}(t)$, and $\hat{\beta}_{AIPW2}(t)$, are given by

```
betahat_Tf
      [,1]  [,2]  [,3]  [,4]  [,5]
[1,] 0.082 0.048 0.035 0.027 0.021
[2,] 0.048 0.048 0.035 0.028 0.021
[3,] 0.035 0.035 0.035 0.028 0.021
[4,] 0.027 0.028 0.028 0.028 0.021
[5,] 0.021 0.021 0.021 0.021 0.021

betahat_IPW
      [,1]  [,2]  [,3]  [,4]  [,5]
[1,] 0.054 0.037 0.029 0.024 0.022
[2,] 0.037 0.036 0.028 0.024 0.022
[3,] 0.029 0.028 0.028 0.024 0.022
[4,] 0.024 0.024 0.024 0.024 0.022
[5,] 0.022 0.022 0.022 0.022 0.022

betahat_AIPW1
      [,1]  [,2]  [,3]  [,4]  [,5]
[1,] 0.049 0.032 0.025 0.021 0.019
[2,] 0.032 0.032 0.025 0.020 0.019
[3,] 0.025 0.025 0.025 0.021 0.019
[4,] 0.021 0.020 0.021 0.020 0.018
[5,] 0.019 0.019 0.019 0.018 0.018

betahat_AIPW2
      [,1]  [,2]  [,3]  [,4]  [,5]
[1,] 0.042 0.029 0.024 0.020 0.019
[2,] 0.029 0.030 0.023 0.020 0.019
[3,] 0.024 0.023 0.024 0.020 0.019
[4,] 0.020 0.020 0.020 0.019 0.018
[5,] 0.019 0.019 0.019 0.018 0.018
```

Under the alternative $\beta_A = 0.405$, the analogous Monte Carlo sample covariance matrices are

```
betahat_Tf
      [,1]  [,2]  [,3]  [,4]  [,5]
[1,] 0.081 0.048 0.035 0.028 0.021
[2,] 0.048 0.048 0.035 0.028 0.022
[3,] 0.035 0.035 0.035 0.028 0.022
[4,] 0.028 0.028 0.028 0.028 0.022
[5,] 0.021 0.022 0.022 0.022 0.022
```

```
betahat_IPW
      [,1]  [,2]  [,3]  [,4]  [,5]
[1,] 0.054 0.037 0.029 0.025 0.023
[2,] 0.037 0.037 0.029 0.024 0.022
[3,] 0.029 0.029 0.029 0.024 0.022
[4,] 0.025 0.024 0.024 0.024 0.022
[5,] 0.023 0.022 0.022 0.022 0.022
```

```
betahat_AIPW1
       [,1]  [,2]  [,3]  [,4]  [,5]
[1,] 0.050 0.033 0.025 0.021 0.019
[2,] 0.033 0.033 0.025 0.021 0.019
[3,] 0.025 0.025 0.026 0.021 0.019
[4,] 0.021 0.021 0.021 0.021 0.019
[5,] 0.019 0.019 0.019 0.019 0.019
```

```
betahat_AIPW2
      [,1]  [,2]  [,3]  [,4]  [,5]
[1,] 0.042 0.029 0.024 0.020 0.020
[2,] 0.029 0.030 0.023 0.020 0.019
[3,] 0.024 0.023 0.024 0.020 0.019
[4,] 0.020 0.020 0.020 0.020 0.019
[5,] 0.020 0.019 0.019 0.019 0.019
```

The foregoing results suggest that the independent increments property continues to hold approximately.

Table C1 presents the estimation results and shows the estimators exhibit little bias under both the null and alternative hypotheses and relative precision similar to that observed in the previous simulations in Section 5 of the main paper where E was taken independent of X.

Table C2 shows that the Type I error is slightly attenuated and the expected sample size and and stopping time under the null and alternative hypotheses are similar to those reported in Section 5 with E independent of X.

In summary, at least in this scenario, the methods appear to be robust to violation of the independence assumption.

TABLE C1 For Scenario 1 with ordered categorical outcome with entry time depending on X; performance of estimators for β under (a) the null hypothesis $\beta = 0$ and (b) the alternative $\beta = \log(1.5) = 0.405$ at each interim analysis time $(t_1, \ldots, t_4) = (150, 195, 240, 285)$ days and at the final analysis at $t_{end} = 330$ days MC Mean is the mean of 10000 Monte Carlo estimates; MC SD is the Monte Carlo standard deviation, Ave MC SE is the mean of Monte Carlo standard errors, and MSE ratio is the ratio of Monte Carlo mean square error for $\widehat{\beta}_{\mathcal{T}_F}(t)$ divided by that for the indicated estimator.

	MC Mean	MC SD	Ave MC SE	MSE ratio	MC Mean	MC SD	Ave MC SE	MSE ratio
				**(a) Null Hypothesis**				
	$\widehat{\beta}_{\mathcal{T}_F}(t)$				$\widehat{\beta}_{IPW}(t)$			
t_1	-0.004	0.286	0.285	1.000	-0.021	0.232	0.229	1.511
t_2	-0.002	0.220	0.219	1.000	-0.024	0.190	0.190	1.311
t_3	-0.002	0.187	0.186	1.000	-0.031	0.168	0.167	1.191
t_4	-0.000	0.167	0.165	1.000	-0.005	0.153	0.153	1.179
t_{end}	-0.000	0.146	0.146	1.000	-0.000	0.147	0.146	0.991
	$\widehat{\beta}_{AIPW1}(t)$				$\widehat{\beta}_{AIPW2}(t)$			
t_1	-0.020	0.222	0.218	1.650	-0.018	0.206	0.199	1.920
t_2	-0.024	0.180	0.180	1.465	-0.018	0.172	0.170	1.615
t_3	-0.031	0.160	0.159	1.316	-0.024	0.154	0.153	1.440
t_4	-0.005	0.143	0 .143	1.355	-0.003	0.139	0.139	1.433
t_{end}	-0.000	0.135	0.135	1.169	-0.000	0.135	0.135	1.169
				**(b) Alternative Hypothesis**				
	$\widehat{\beta}_{\mathcal{T}_F}(t)$				$\widehat{\beta}_{IPW}(t)$			
t_1	0.411	0.284	0.284	1.000	0.391	0.233	0.230	1.491
t_2	0.410	0.219	0.218	1.000	0.386	0.192	0.191	1.293
t_3	0.409	0.187	0.186	1.000	0.377	0.169	0.168	1.191
t_4	0.410	0.167	0.16	1.000	0.399	0.155	0.154	1.170
t_{end}	0.406	0.147	0.146	1.000	0.406	0.148	0.147	0.985
	$\widehat{\beta}_{AIPW1}(t)$				$\widehat{\beta}_{AIPW2}(t)$			
t_1	0.391	0.223	0.219	1.628	0.402	0.205	0.199	1.929
t_2	0.386	0.1821	0.180	1.446	0.399	0.172	0.170	1.625
t_3	0.377	0.161	0.160	1.315	0.392	0.155	0.153	1.460
t_4	0.399	0.144	0.144	1.343	0.408	0.141	0.139	1.418
t_{end}	0.406	0.137	0.136	1.159	0.406	0.137	0.136	1.159

TABLE C2 For Scenario 1 with ordered categorical outcome with entry time depending on X; interim analysis performance using each estimator with O'Brien-Fleming and Pocock stopping boundaries under (a) the null hypothesis $\beta = 0$ and (b) the alternative $\beta = \log(1.5) = 0.405$, with maximum sample size $n_{max} = 602$ and $t_{end} = 330$ days. P(reject) is the proportion of Monte Carlo data sets for which the null hypothesis was rejected; MC E(SS) is the Monte Carlo average of number of subjects enrolled at the time the stopping boundary was crossed (standard deviation); and MC E(Stop) is the Monte Carlo average stopping time (days) (standard deviation). The standard error for entries for P(reject) in (a) is ≈ 0.0016.

	**P(reject)**	**MC E(SS)**	**MC E(Stop)**	**P(reject)**	**MC E(SS)**	**MC E(Stop)**
			**(a) Null Hypothesis**			
	**O'Brien-Fleming**			**Pocock**		
$\widehat{\beta}_{T_F}(t)$	0.024	601.9 (4.5)	329.3 (7.6)	0.024	599.1 (24.4)	327.1 (21.0)
$\widehat{\beta}_{IPW}(t)$	0.022	601.7 (6.6)	328.9 (9.7)	0.021	599.1 (25.1)	327.3 (20.8)
$\widehat{\beta}_{AIPW1}(t)$	0.024	601.6 (7.8)	328.8 (10.3)	0.021	599.0 (25.5)	327.2 (21.1)
$\widehat{\beta}_{AIPW2}(t)$	0.022	601.3 (11.4)	328.3 (13.0)	0.022	598.4 (27.8)	326.8 (22.9)
			**(b) Alternative Hypothesis**			
	**O'Brien-Fleming**			**Pocock**		
$\widehat{\beta}_{T_F}(t)$	0.786	588.5 (40.6)	284.5 (47.0)	0.711	541.7 (90.4)	259.6 (70.5)
$\widehat{\beta}_{IPW}(t)$	0.765	564.3 (67.6)	264.7 (56.9)	0.683	518.0 (102.4)	245.8 (76.7)
$\widehat{\beta}_{AIPW1}(t)$	0.829	558.5 (71.0)	257.0 (55.9)	0.755	509.4 (103.5)	237.0 (75.1)
$\widehat{\beta}_{AIPW2}(t)$	0.826	529.9 (87.4)	237.3 (60.6)	0.763	485.2 (106.3)	220.6 (75.0)
